# In Silico Studies of Lamiaceae Diterpenes with Bioinsecticide Potential against *Aphis gossypii* and *Drosophila melanogaster*

**DOI:** 10.3390/molecules26030766

**Published:** 2021-02-02

**Authors:** Gabriela Cristina Soares Rodrigues, Mayara dos Santos Maia, Andreza Barbosa Silva Cavalcanti, Natália Ferreira de Sousa, Marcus Tullius Scotti, Luciana Scotti

**Affiliations:** 1 Laboratory of Cheminformatics, Program of Natural and Synthetic Bioactive Products (PgPNSB), Health Sciences Center, Federal University of Paraíba, João Pessoa, PB 58051-900, Brazil; gaby.ecologia@gmail.com (G.C.S.R.); mayarasmaia@hotmail.com (M.d.S.M.); andreza.jp.pb@gmail.com (A.B.S.C.); nferreiradesousa.nfs@gmail.com (N.F.d.S.); mtscotti@gmail.com (M.T.S.); 2 Health Sciences Center, Lauro Wanderley University Hospital, Federal University of Paraíba, João Pessoa, PB 58059-900, Brazil

**Keywords:** bioinsecticides, Lamiaceae, diterpenes, virtual screening, machine learning, docking

## Abstract

Background: The growing demand for agricultural products has led to the misuse/overuse of insecticides; resulting in the use of higher concentrations and the need for ever more toxic products. Ecologically, bioinsecticides are considered better and safer than synthetic insecticides; they must be toxic to the target organism, yet with low or no toxicity to non-target organisms. Many plant extracts have seen their high insecticide potential confirmed under laboratory conditions, and in the search for plant compounds with bioinsecticidal activity, the Lamiaceae family has yielded satisfactory results. Objective: The aim of our study was to develop computer-assisted predictions for compounds with known insecticidal activity against *Aphis gossypii* and *Drosophila melanogaster*. Results and conclusion: Structure analysis revealed *ent*-kaurane, kaurene, and clerodane diterpenes as the most active, showing excellent results. We also found that the interactions formed by these compounds were more stable, or presented similar stability to the commercialized insecticides tested. Overall, we concluded that the compounds bistenuifolin L (**1836**) and bistenuifolin K (**1931**), were potentially active against *A. gossypii* enzymes; and salvisplendin C (**1086**) and salvixalapadiene (**1195**), are potentially active against *D. melanogaster*. We observed and highlight that the diterpenes bistenuifolin L (**1836**), bistenuifolin K (**1931**), salvisplendin C (**1086**), and salvixalapadiene (**1195**), present a high probability of activity and low toxicity against the species studied.

## 1. Introduction

The growing demand for agricultural products has led to the misuse of insecticides; resulting in the use of higher concentrations and the need for ever more toxic products [1]. This has led to increases in toxic effects on other beneficial organisms, (those which coexist with pests within the agroecosystem), in bioaccumulation of toxic insecticide concentrations in the bodies of both predators and endpoint consumers, including humans [2,3,4]. Most of the available insecticides for crop use are synthetic and often persist as toxic waste in the environment far beyond the time desired, thus causing both organismal resistance and environmental pollution [5,6].

The undesirable effects of using chemical pesticides include resistance in pests, pollution, and acute and chronic health problems [7,8,9]. The growing concern about environmental and health risks from the use of pesticides has led to prohibition of many traditional pesticides and substitutions with less toxic compounds [10,11,12]. Plant products are a rich source of active compounds with potential as insecticides, antifeedants, antimoulting hormones, oviposition impediments, repellents, juvenile hormone simulators, and growth inhibitors; and presenting promise against specific target insects [13,14].

Bioinsecticides, to be considered ecologically correct and safer than synthetic insecticides, must be toxic to the target organism with little or no toxicity to the non-target organism [14,15,16]. Many plant extracts present confirmed high insecticide potential in the laboratory [15,17,18,19], yet the lack of studies on mechanisms of action diminishes their potential for large-scale application [2,20].

In the search for compounds with bioinsecticidal activity, the family Lamiaceae has brought satisfactory results [21,22,23]. Lamiaceae has a cosmopolitan distribution with approximately 295 genera and 7775 species [24,25,26]. It is divided into 12 subfamilies, with Nepetoideae being the largest, containing almost half of the genera and species. Nepetoideae presents many compounds with great structural diversity, including the diterpenes commonly reported [26].

In this work, *Aphis gossypii* and *Drosophila melanogaster* were studied, insects causing significant harm to agriculture and to man. *Aphis gossypii* (Hemiptera: Aphididae) is found in many countries: India, China, the United States, Brazil, Pakistan, Australia, and Turkey, and is a global agricultural pest that affects cotton crops worldwide [27,28,29]. The pest damages many plant species worldwide causing significant economic losses, including in the families of Cucurbitaceae (melon, marrow, zucchini, watermelon) [27,30,31], and Solanaceae (chili and tomato), and in various ornamental flower species [31,32].

*Aphis gossypii* causes serious economic losses through direct feeding, virus transmission, and contamination [33], these can quickly damage plants, since aphids have a short reproductive cycle, and under the appropriate environmental conditions, great reproductive capacity [34]. *A. gossypii* control is performed using insecticides, yet the insect has developed resistance to a variety of insecticides including organophosphates, pyrethroids, and neonicotinoids [33,35].

The common fruit fly, *Drosophila melanogaster* (Diptera: Drosophilidae) has an average life-span of from 50 to 60 days [36]. It is associated with sour rot in ripe or damaged grapes causing serious damage to viticulture [37,38,39]. *Drosophila melanogaster* is a model organism in toxicological studies and for testing insecticide activity [10,40,41,42,43,44,45]. The species has an easily manipulated genome that enables generation of pathway mutations, which can assist in understanding factors that influence insecticide functionality [41,46,47].

Although *Drosophila melanogaster* is not considered an agricultural pest, as it does not cause extensive crop damage, its presence in households and consumer market places remains a problem; indicating either unhealthy fruit or a poor environment [36]. *Drosophila melanogaster* causes damage to different types of pulp fruits, mainly guava and bananas [23], and controlling fruit flies is an important factor in both the global economic and in public health [13]. Synthetic insecticides, though still widely used, promote most insect pests to acquire resistance [48,49,50].

To evaluate compounds with potential insecticidal activities, Virtual Screening (VS) is used to select compounds with desired properties by screening chemical compound libraries with computational models [51,52,53], as well as evaluating compounds which are potentially dangerous to the environment [4,53,54,55,56,57]. The aim of our study was to develop computer-assisted predictions for compounds presenting known insecticidal activity against *Aphis gossypii* and *Drosophila melanogaster*.

### 1.1. Chitinase

Chitinase (Cht) enzymes belong to the glycosyl hydrolases (GH) group; families GH18 and GH19; family GH20 contains additional chitinolytic enzymes [58]. These include beta-N-acetylhexosaminidases, generally called chitobiases, which catalyze decomposition of dimeric GlcNAc units (chitobiose) into monomers from chitin or chitin-dextrin terminal reducing ends [59]. Although all of these enzymes hydrolyze beta-(1,4) glycosidic bonds of acetylated D-glucosamine units, there are substantial differences in their modes of action, amino acid sequences, and catalytic sites. While the catalytic regions of the GH18 and GH20 glycosidases are characterized by a barrel (β/α) 8 triosephosphate isomerase (TIM) domain, the GH19 chitinases have a lysozyme-like domain, rich in α-helices [60,61]. In insects, chitinases belong to the GH18 family which promotes to rapid depolymerization of the chitin polymer in insects [62,63,64].

#### 1.1.1. *Drosophila melanogaster*

Chitinase involvement in the activity of insecticides is reported by Zhang et al. (2018) [10], where the authors investigated the action of the substance Azadirachtin, a botanical terpene insecticide, derived from *Azadirachta indica* A. Juss (Meliaceae); the Neem tree. The methodology evaluated survival, growth, and reproduction metrics in *Drosophila melanogaster*; survival tests of adults, evaluation of oxidative stress levels, and evaluation of chitinase and caspase activity, as well as occurrences of apoptosis.

Azadirachtin at 10 mgL^−1^ induced the death of *D. melanogaster* after 3 to 7 days of exposure, and at the dose of 20 mgL^−1^ death was induced after 5 days of continuous exposure. Further, the compound appears to have less pronounced toxicity (LD_50_ = 630 mg L^−1^, LD_50_ = 670 mg L^−1^) when applied topically to adult fruit flies or larvae, indicating that susceptibility to Azadirachtin varies according both application method and time of exposure. The molecular mechanisms of acute toxicity of ingested Azadirachtin are unclear, but studies in *D. melanogaster* larvae have shown that it appears to affect mainly post-transcriptional enzyme regulation, proteins involved in cytoskeleton development and transcription, translation and regulation of hormones and energy metabolism, and in general, Azadirachtin reduces *D. melanogaster*’s life-span.

The authors found lower chitin levels, and higher chitinase levels with exposure to Azadirachtin, indicating that Azadirachtin-induced growth inhibition may be closely associated with chitin levels. This demonstrates the influence of Azadirachtin on chitin synthesis, and also that its inhibitive effects are regulated by the expression of the chitin synthase gene, which is vital to maintaining the exoskeleton, as well as to growth and organ remodeling.

The authors concluded that Azadirachtin intake at 4 mg L^−1^ causes a series of sub-lethal effects in *D. melanogaster*, affecting longevity, development, and reproduction; due to interference in various endocrinological and physiological functions.

Similarly, Bezzar-Bendjazia (2017) [65] also evaluated inhibition of chitinase using Azadirachtin, but with the objective of preventing larval evasion, and expression of digestive enzymes. The authors demonstrated that chitinase activity decreased in treated larvae when compared to the controls (F2, 15 = 202.4; *p* < 0.001). The mean values recorded were 0.64 ± 0.009 mmol/min/mg of protein for the controls, 0.51 ± 0.005 mmol/min/mg of protein for LD25, and 0.44 ± 0.006 mmol/min/mg of protein for the LD_50_. Statistical analysis revealed a significant difference between the two doses tested (*p* < 0.05). Treatment of *D. melanogaster* larvae with Azadirachtin significantly reduced α-amylase, chitinase, and protease activities in the intestine, and increased lipase activity. Azadirachtin affects larval evasion, food consumption, and digestion in *D. melanogaster*; suggesting it as a promising insecticide.

Chitinase inhibition has also been reported by Kilani-Morakchi and collaborators (2017) [66]. In this study, the authors assessed the difference in the levels of enzyme expression between males and females after application of Azadirachtin to evaluate the mechanisms of food selection and expression of digestive enzymes. The analyses revealed significant dose based effects (F2, 30 = 48.81; pb0.001) (pb0.001), sex based effects (F1, 30 = 44.94; pb0.001), and dose–sex interaction effects (F2, 30 = 8.67; pb0.001). The mean values for the control series were 0.440 ± 0.010 mmol/min/mg of proteins, and 0.540 ± 0.012 mmol/min/mg of proteins, respectively, for males and females. In the treated series, the mean values recorded were 0.420 ± 0.009 mmol/min/mg of proteins for LD_25_ and 0.370 ± 0.005 mmol/min/mg of proteins for LD_50_ for males, and 0.430 ± 0.012 mmol/min/mg of proteins for LD_25_ and 0.420 ± 0.010 mmol/min/mg of proteins for LD_50_ for females. As a result, the authors concluded that the bioinsecticide affects the activity of digestive enzymes in the lower-extremities of the intestine. The results may reflect Azadirachtin’s interference in food and metabolism regulation, and provide evidence of delayed effects at this stage of development; reinforcing its insecticidal activity.

Another study developed by Loper and Collaborators (2016) [67], with strains of *Pseudomonas fluorescensis*, aimed to evaluate production of toxic mediators in *D. melanogaster*; such as Pf-5, Rizoxima, and Orfamida A. The methodology employed gene expression, with morphological, and phylogenetic analyses. The authors concluded that the oral toxicity induced by *Pseudomonas fluorescensis* by means of the Pf-5 gene was significant, being induced in several genes and that the effect is promoted by an extracellular chitinase and Rhizoxin and Orfamide A analogs.

#### 1.1.2. *Aphis gossypii*

For *Aphis gossypii*, only 8 articles deal with the topic. Elbanhawhy and Collaborators (2019) [62], addressed the insecticidal activity of different organic extracts from entomopathogenic fungi; *Cladosporium cladosporioides, Metarhizium anisopliae, Purpureocillium lilacinum,* and *Trichoderma longibrachiatum* against the cotton aphid, *Aphis gossypii*. The impact of the extracts on certain biochemical characteristics and enzyme activity was also evaluated.

The authors performed extraction of metabolites from the fungus, as well as toxicity tests, evaluation of total carbohydrates and triglycerides as effects, and evaluation of enzymatic activity. The results revealed that there was a significant increase (*p* < 0.05) in chitinase activity, respectively of 34.85% and 9.82%, for the *C. cladosporioides* and *P. lilacinum* extracts under study.

The authors concluded that chitinase plays an important role in the growth and development of insects, in addition they reported that this family of enzymes is involved in insect defense against entomopathogenic fungi, since chitinase activity increased after treatment with *P. lilacinum* methanolic extract. The extract thus can be used in chitin biodegradation, which leads to the death of the aphid.

The use of alcoholic derivatives was explored by Kim and Collaborators (2013) [68], the authors evaluated the effect of isotridecyl alcohol ethoxylation on the larvicidal activity of fungal supernatant, where the fungus was isolated and the supernatant was produced. To achieve the proposed objective, the authors determined aphicidal activity using the leaf immersion method, which suggested that the performance of the supernatant may be related to the activity of the enzymes chitinases and lipases, since they participate in the degradation of the cuticle by fungi and entomopaths. This enzymatic degradation was possibly improved by the TDE-less ethoxylate, being noted that at each ethoxylation a synergistic interaction between the supernatant and the TDE-n was evidenced.

Thus, the authors concluded that the supernatant *B. bassiana* (SFB-205) with less ethoxylated tridecyl alcohol (TDE) presents greater insecticidal activity against cotton aphid adults. The incorporation of TDE into the supernatant increased the potency of the fungal supernatant in an ethoxylation dependent manner. This finding represents a practical approach to effectively control harmful agricultural insects using an entomopathogenic fungal supernatant or spores (conidia).

In another study Kim and Collaborators (2010) [69], evaluated the expression of enzymes for pest control through a study with fungi, which evaluated aphicidal activity in expression of *Beauveria bassiana* enzymes, this being their initial study. The work aimed to describe the roles of adjuvants, such as corn oil, and polyoxyethylene-(3)-isotridecyl ether (TDE-3), in promoting the aphicidal activity of the enzymatic precipitate of *Beauveria bassiana* SFB-205 supernatant. The methodology consisted of isolating the fungus, preparing the suspension, and screening.

Regarding chitinase degradation, the authors reported its occurrence and variance under different conditions, with the AMEP + TDE-3 suspension (based on corn oil) degrading the specific chitinase substrate more pronouncedly than the AMEP + TDE-3 water-based suspension in dry conditions. Compared to unexposed suspension, 73% of the substrate (pNG) was degraded by treatment with the AMEP + TDE-3 suspension based on corn oil, 120 min after drying. However, the AMEP + TDE-3 water-based suspension treatment yielded only 18.5% pNG degradation for the same exposure time. Degradation was significantly affected by suspension in water or corn oil (base type (B): F1.72 = 15.9, Pb0.001; exposure time (E): F5.72 = 1.8, *p* = 0.133; and B × E: F5.72 = 4.1, *p* = 0.032). In addition, treatment with the AMEP + TDE-3 suspension based on corn oil, a large part of the suspension remained in the wells even after incubation for 120 min. On the other hand, little or no remaining suspension was observed in with the AMEP + TDE-3 water suspension treatment at the end of the exposure.

Thus, the authors conclude that corn oil with TDE-3 can promote the insecticidal activity of attagel-mediated enzyme powder (AMEP), to provide another strategy for the development of biopesticides using entomopathogenic fungi. Kim and collaborators (2010) [70], evaluated the expression of *B. bassiana* enzymes to control *Aphis gossypii*, in two FPLC fractions. Chitinase activities were expressive and identified for the fractions, corresponding to 55 KDa of the protein band. The authors concluded that the two FPLC fractions from *Beauveria bassiana* SFB-205 supernatant, displaying chitinase or Pr1/Pr2 protease activity and bioassayed against *Aphis gossypii* in different ratios, promoted a decrease in the aphid population, yet this decrease was more significantly influenced by the chitinase fraction (in a dosage-dependent manner).

### 1.2. Acethylcolinesterase

Acetylcholinesterase (AChE) inhibition is the main mechanism of action of organophosphates [71,72,73]. The enzyme is essential and necessary for hydrolysis of the neurotransmitter acetylcholine (ACh), and plays a fundamental role in controlling synaptic transmission in all animals; hydrolyzing acetylcholine to end its synaptic action [74,75,76].

The enzyme AChE belongs to the group of phase I metabolic enzymes and can metabolize various internal and external substrates in pests; this group of metabolic enzymes consists of broad spectrum enzymes capable of metabolizing chemical insecticides such as organophosphates, carbamates, or pyrethroids [77,78]. Increasing or decreasing the amount of these enzymes leads to loss of efficiency in insecticides; thus, agents with new and different mechanisms of action must be developed for insect control [79,80,81,82].

#### 1.2.1. *Drosophila melanogaster*

In studies involving *Drosophila melanogaster*, the participation of acetylcholinesterase is widely reported, on average 400 publications on the subject are identified, and the studies use various methodologies.

In a computational study of Quantitative Chemical Structure and Biological Activity Relationship (QSAR), Rodrigues and Collaborators (2020) [83], evaluated the potential insecticidal activity of monoterpenes against the insects *Reticulitermes chinensis* and *Drosophila melanogaster*. Construction of linear regression models was performed in which the activity was expressed in pIC_50_ which is equivalent to −log LC_50_. The descriptors were obtained using the Dragon software version 7.0 and the selection of variables for later calculation by the genetic algorithm was performed in the Mobydigs 1.1 software using the multiple linear regression equation (MLR) method. Only the models with the highest Q^2^ values were selected according to workflows. Molecular Docking simulations were performed using the Molegro Virtual Docker 6.1.0 software, with the proteins obtained from the Protein Data Bank PDB library. One of the proteins chosen was acetylcholinesterase under code: 1QON, 2.7 Å resolution, using a 15 Å GRID in the radius, and 0.30 Å resolution at the enzyme binding site with the structures.

The results revealed that the 40 monoterpenes present interaction values very close to known insecticides. Neryl acetate presented the lowest energy at −87 kcal/mol, close to the insecticides methiocarb and pirimicarb (carbamates), with the energies of −90 kcal/mol. The compound neryl acetate was one of the most active in the series, followed by citronellyl acetate with −83 kcal/mol, geranyl acetate with −78 kcal/mol and linalyl acetate with −77 kcal/mol; monoterpenes presenting the lower energies. In addition, the compounds presented significant interactions with the enzyme, suggesting that the monoterpenes belonging to the acetates group interact more strongly with acetylcholinesterase, and possibly that other monoterpene groups may present different mechanisms of action. The stability of the interaction was verified by molecular dynamics simulations revealing that the stability of the AChE active site was guaranteed by formation of complexes with three selected terpenes, being comparable to pirimicarb and methiocarb.

The authors concluded that pulegone, citronella, carvacrol, linalyl acetate, neryl acetate, citronella acetate, and geranyl acetate can be considered potential pesticide candidates.

In another study Gomes and collaborators (2020) [75], evaluated the insecticidal action of *Croton campestris* methanolic extract, and the protective effect of gallic acid. The methodology used consisted of quantification of compounds by HPLC, enzymatic and locomotion assay, and evaluation of enzyme expression (acetylcholinesterase). The results showed that organophosphates reduced the expression of acetylcholinesterase by 66%, but this result was blocked by the study compound MFCC. The action was also observed in other enzymes such as superoxide dismutase.

The authors concluded that chemical constituents of the plant prevented organophosphate induced AChE inhibition, and attributed essential neuroprotective potential to the plant. It was shown that gallic acid contributes to the fraction’s protective potential as compared to other phenolic compounds. Thus, MFCC was considered a promising source of potential therapeutic agents for the treatment of organophosphate intoxication.

Similarly, Abbod (2020) [84], analyzed the mode of action of the substance 3-butylidenephthalide, a natural pesticide. Their results showed that the study compound (3-BPH) exhibited in vitro activity for i-AChE in a concentration-dependent manner; the percentage of inhibition of the enzyme varied respectively between 14% ± 3.21 to 69% ± 4.93 for 50 μg/mL to 1000 μg/mL (mean ± SE of the three repetitions). Physostigmine was used as a standard AChE inhibitor and revealed an IC_50_ of 0.082 μg/mL. Thus, the authors suggested 3-BPH as an important plant protector.

Musachio and collaborators (2020) [85], demonstrate the development of Parkinson’s in *Drosophila melanogaster* species through exposure to Bisphenol A (BPA). Their results reveal an association with acetylcholinesterase levels, since in the head samples, there was a decrease in the activity of the enzyme AChE in both groups exposed to BPA (at 0.5 mM and 1 mM), when compared to the control group (Ap < 0.0007; F = 18.08). However in the body samples, the activity of AChE did not change (Bp < 0.2738; F = 1.620). The authors concluded that bisphenol induces changes similar to Parkinson’s, and is possibly associated with oxidative stress, suggesting new options for future study.

#### 1.2.2. *Aphis gossypii*

For the involvement of acetylcholinesterase in *Aphis gossypii*, 88 articles were found.

Authors Ulusoy, Özgür and Alpkent (2019) [80], reported on the effect of in vitro anti-acetylcholinesterase and anti-carboxylesterase toxicity for various plant extracts. The plants used in the test were: *Daphne odora* L. *(Malvales: Daphne), Dieffenbachia amoena* L. *(Alismatales: Thymelaeaceae), Eucalyptus camaldulensis* L. *(Myrtales: Myrtaceae), Ficus carica* L. *(Rosales: Moraceae), Lantana Câmara* L. *(Lamiales: Verbenaceae), Matricaria chamomilla* L. *(Asterales: Asteraceae), Mentha pulegium* L. *(Lamiales: Lamiaceae),* and *Nerium oleander* L. *(Gentianales: Apocynoideae)*.

The methodology consisted of preparing extracts of the plants and performing toxicity tests, evaluating the effects of inhibiting acetylcholinesterase and carboxyesterase. The results demonstrated that *F. carica* extract in all concentrations was the most effective in inhibiting the expression of acetylcholinesterase. *Ficus carica* presented the greatest AChE inhibitory effect (51.9% inhibition). The least AChE inhibitory effect was 10% with *D. amoena* (20.9% inhibition). The most effective plant extracts after *F. carica* were *D. odora* (41.0% AChE inhibition), and *E. camaldulensis* (40.3% AChE inhibition). In conclusion, it was determined that aqueous *D. odora, E. camaldulensis, F. carica*, and *M. pulegium* leaf extracts present significant bioinsecticide effect and in vitro anti-AChE activities in *A. gossypii*.

The influence of acetylcholinesterase expression is demonstrated through studies of resistance associated with pirimicarb. Research developed by Tieu and Collaborators (2017) [86] found that pyrimidine resistance presented a significant cost in physical conditioning in susceptible aphids and in the absence of insecticidal pressure, and that the cost of physical conditioning was related to initial resistance, due to the involvement of acetylcholinesterase receptors.

The occurrence of mutation in acetylcholinesterase receptors in the melon aphid through exposure was also addressed by Lokeshwari, Kumar, and Manjunatha (2016) [87]. The AChE enzyme assay revealed that there was no significant change in AChE activities in resistant and susceptible strains. However, the AChE inhibitory assay revealed that 50% of enzymatic activity in resistant strains was inhibited at significantly higher concentrations of dimethoate (131.87, 158.65, and 99.29 µmol L^−1^) compared to susceptible strains (1.75 and 2.01 µmol L^−1^), indicating insensitivity to AChE due to AChE modulation.

Functional analysis of such point mutations was evaluated in molecular docking studies, using modeled wild type and naturally mutated AChE2. Computational analysis showed that conformational changes in the AChE2 active site due to structural gene substitutions (A302S, S431F, and G221A) significantly reduced the level of ligand binding (OP-dimethoate, Omethoate, and CM-pirimicarb), suggesting that they are potentially associated with resistance development.

The authors concluded that multiple mutations located at the active site of the enzyme are responsible for AChE insensitivity to dimethoate, and are probably the molecular basis of resistance to dimethoate in these *A. gossypii* populations. In addition to studies on the occurrence of mutations, there are also studies involving proteomic profiling, and protein analysis; such as research carried out by Xi and Collaborators (2015) [88], involving Spirotetramat tolerance, where the authors demonstrated that acetylcholinesterase conferred resistance to the substance under study.

### 1.3. Nicotinic Acetylcholine Receptor

**Figure 1:**
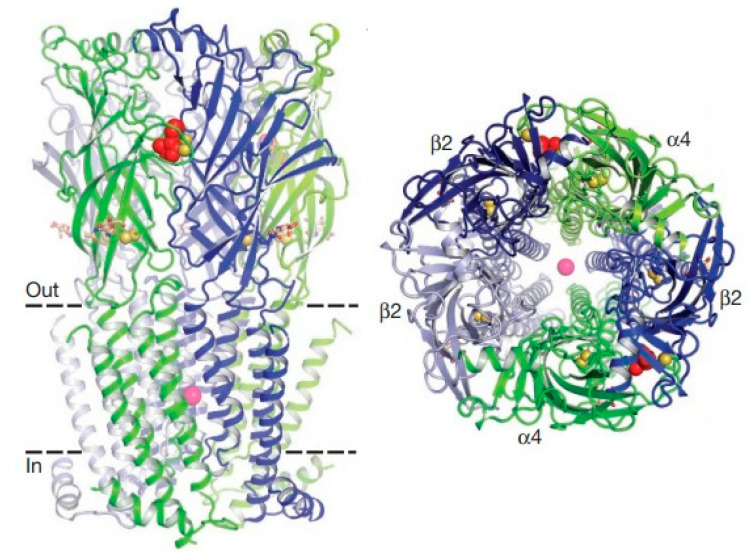
Structure of the nicotinic acetylcholine receptor. Source: Moralles-Perez, Noviello, Hibbs (2016) [97].

Acetylcholine has two types of receptors, which are classified into nicotinic (nAChRs) and muscarinic (mAChRs) receptors [89,90,91]. Nicotinic receptors present as ion channels dependent on pentameric ligands that are activated by acetylcholine, as well as by nicotine to trigger action potentials for rapid synaptic neurotransmission [92]. In mammals, nicotinic receptors are expressed in presynaptic regulation to regulate the release of dopamine and other neurotransmitters from the nigrostriatal terminals. In insects, mainly in *Drosophila melanogaster* species, nicotinic receptors are expressed in abundance throughout the central nervous system [93,94,95,96]. Figure 1 demonstrates the structure of the nicotinic acetylcholine receptor [97]. The receptor is pentameric. The α4 subunits are green, and β2 is blue. Nicotine (red) and sodium (pink) are represented as spheres. The disulfide Cys-loop and C-loop connections are shown as yellow spheres.

#### 1.3.1. *Drosophila melanogaster*

For *Drosophila melanogaster*, about 230 articles were found related to the nicotinic acetylcholine receptor.

Research developed by Fournier-Level and Collaborators (2019) [98], addressed the expression of receptors in *Drosophila melanogaster* after exposure to imidacloprid. Population and quantitative genomic analysis, supported by functional tests, revealed a mixed genetic architecture for resistance involving the main genes (Paramyosin, and Receptor Nicotinic-Acetylcholine Alpha 3), and polygenes with a large exchange and thermo-tolerance. The reduced genetic differentiation in the sites associated with resistance indicated an increase in gene flow. Resistance alleles showed stronger evidence of positive selection in temperate populations as compared to tropical populations, in which chromosomal inversions In (2L) t, In (3R) Mo, and In (3R) Payne harbor susceptibility alleles.

Thus, the authors concluded that polygenic architecture and ecological factors should be considered when developing sustainable management strategies for beneficial pests and insects.

Similarly, Shin and Ventom (2018) [96], evaluated the electrochemical mechanisms of Dopamine receptor stimulation in *Drosophila melanogaster*. In this study, acetylcholine and nicotine were used as stimulants, since both interact with nicotinic acetylcholine receptors (nAChRs) to evoke endogenous dopamine release. Stimulation with 10 pmol acetylcholine caused 0.26 ± 0.05 μM dopamine release, while nicotine stimulation at 70 fmol evoked 0.29 ± 0.03 μM dopamine release in the central complex. The release of dopamine stimulated by nicotine lasted much longer than the release stimulated by acetylcholine. Dopamine release is reduced in the presence of nAChR α-bungarotoxin antagonist, and the sodium channel blocker tetrodotoxin, indicating that release is mediated by nAChRs and exocytosis.

Another mechanism studied is the involvement of serine metabolism in the hunger regulation and sleep modulation in *Drosophila melanogaster*, developed by Sonn and collaborators (2018) [99]. Their results revealed that mutation in the stdh gene in the acetylcholine nicotinic receptor selectively rescued the suppression of sleep induced by exaggerated hunger, through the administration of an antagonist. Thus, the authors conclude that neural serine metabolism controls sleep during hunger, possibly via cholinergic signaling, due to the development of a sleep regulatory mechanism that reprograms the metabolism of amino acids for adaptive sleep behaviors in response to metabolic needs.

Exposure to nicotine in larval development through activation of dopamine receptors in *Drosophila melanogaster* was addressed by Morris and collaborators (2018) [100]. The authors demonstrated the involvement of the Dα7 subunit of nicotinic acetylcholine receptors in the early hatching of larvae, and concluded that exposure to nicotine during *Drosophila melanogaster* development affects the size of the brain and the dopaminergic system.

#### 1.3.2. *Aphis gossypii*

To demonstrate the involvement of the nicotinic acetylcholine receptor in the action of insecticides against *Aphis gossypii*, 30 articles were found.

Of the methodologies covered, analysis of receptor mutation was performed by Hirata and Collaborators (2017) [101], evaluating mutation of the R81T gene. This mutation is characterized as the source of resistance to neonicotinoid insecticides. The authors evaluated the differential effects of the R81T mutation in cyan and nitro-substituted neonicotinoids and in sulfoxaflor, and for this purpose, isolation of the complete coding sequences for A. *gossypii* in the AChRα1, α2, β1 subunits was performed.

The results revealed that when co-expressed in *Xenopus laevis* oocytes in chicken β2 nAChR, *A. gossypii* α1 evoked internal currents in a concentration-dependent manner in response to acetylcholine (ACh) and showed sensitivity to neonicotinoid and sulfoxaflor. In addition, the chicken β2 mutation T77R + E79V (double mutant equivalent of R81T) resulted in a lesser effect on cyano-substituted neonicotinoids and sulfoxaflor than on nitro-substituted neonicotinoids (neonicotinoid insecticides replaced by cyan and nitro).

The authors concluded that the R81T mutation presents resistance to nicotinoids in nAChRs, and the mutation affects distinctly cyan and nitro-substituted neonicotinoids.

The occurrence of mutations in the nicotinic receptor was also addressed by Chen and Collaborators (2017) [102]. The authors identified three mutations at the target site within the β1 subunit of the nicotinic acetylcholine receptor (nAChR) in the IMI_R strain, with the R81T mutation being responsible for imidacloprid resistance in *A. gossypii* and *M. persicae*. The V62I and K264E genes were first detected in *A. gossypii*. The mutations are also present in internal populations, suggesting that they play a role in resistance to imidacloprid.

Other articles that address resistance after the occurrence of mutations in subunits of the nicotinic acetylcholine receptor, comprise research developed by Toda and Collaborators (2017) [103], studying a point mutation (R81T) in the region of the D loop of the β1 subunit of the acetylcholine nicotinic receptor gene conferring resistance, and identification of neonicotinoid resistance using the Polymerase Chain Reaction (PCR) methods.

The mutation in the R81T gene has also been reported by Wang and Collaborators (2016) [104]; however, their objective was α2 and β1 subunits after administration of Sulfoxaflor. The results demonstrated that the van der Waals interactions of whole molecules were highly correlated with neonicotinoid binding capacity, and correctly predict the classification order of the association between neonicotinoids and sulfoxaflor. Further, changes in a whole molecule electrostatic energy component can potentially explain the effects of the mutation at the target site through a pattern of reduced efficacy for modeled neonicotinoids, and provide a basis for reducing the effect of this mutation on sulfoxaflor.

## 2. Results and Discussion

### 2.1. Protein Sequence Alignment

Protein sequence alignment helps to verify similarity and identity of a single protein in different species. Using this technique, one can analyze conserved regions and identify common residues in the active site. In addition, structural differences and similarities that can contribute to the development of drugs may be revealed. Thus, we investigated shared amino acids from AChE, nAChR, and Cht sequences in *A. gossypii* and *D. melanogaster*.

**Figure 2:**
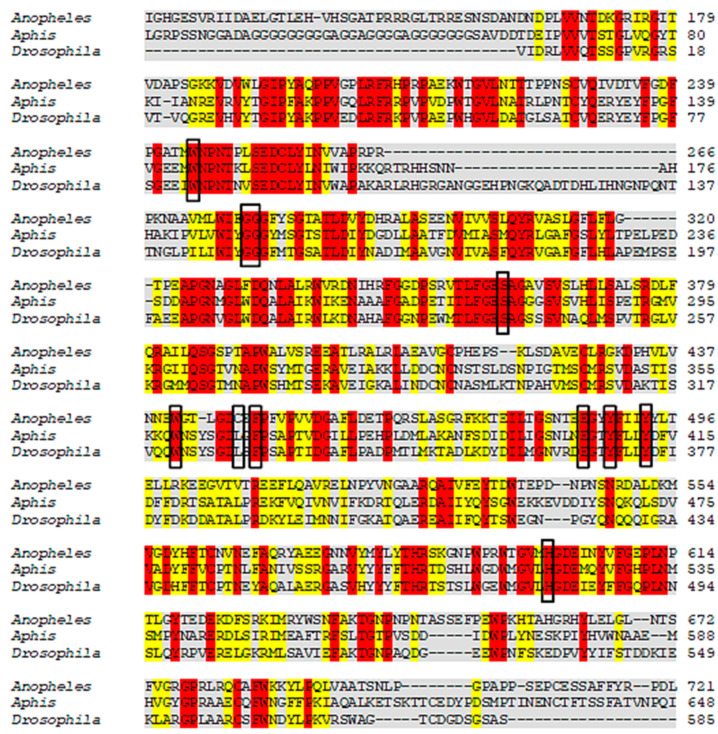
Alignment of *Anopheles gambiae*, *Aphis gossypii,* and *Drosophila melanogaster* Acetylcholinesterase (AChE) protein sequences. The gray regions represent non-similar and non-identical amino acids. The red regions represent identical amino acids. The yellow regions represent similar amino acids. The black boxes represent conserved regions of the active site which bind to the inhibitor.

**Figure 3:**
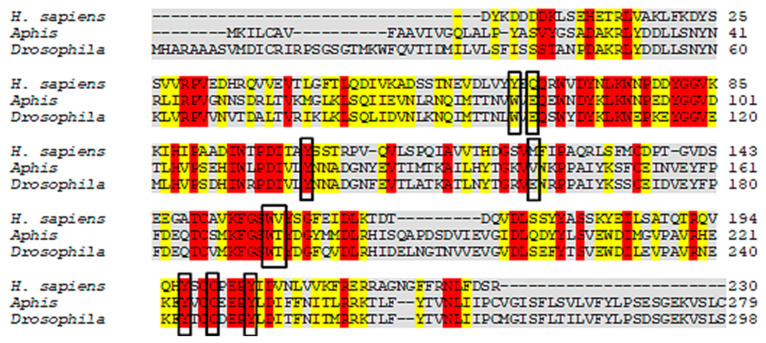
Alignment of *H. sapiens*, *A. gossypii,* and *D. melanogaster* nicotinic acetylcholine receptors (nAChRs) protein sequences. The gray regions represent non-similar and non-identical amino acids. The red regions represent identical amino acids. The yellow regions represent similar amino acids. The black boxes represent conserved regions of the active site which bind to the inhibitor.

**Figure 4:**
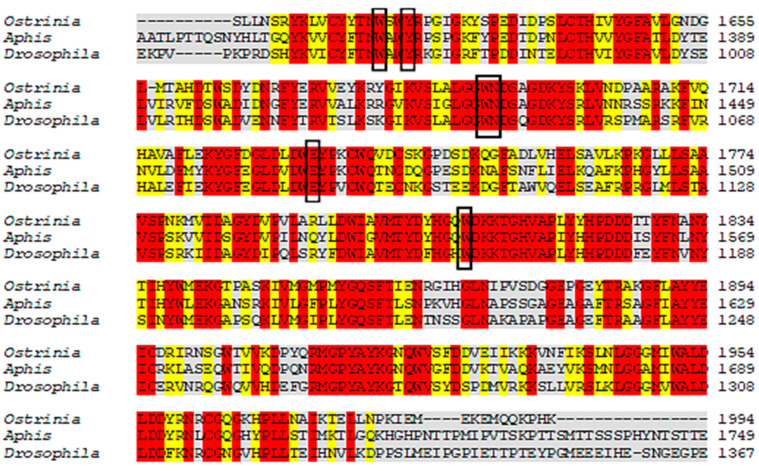
Alignment of *Ostrinia furnicalis*, *A. gossypii,* and *D. melanogaster* Cht protein sequences. The gray regions represent non-similar and non-identical amino acids. The red regions represent identical amino acids. The yellow regions represent similar amino acids. The black boxes represent conserved regions of the active site which bind to the inhibitor.

The results revealed that *A. gossypii* and *D. melanogaster* respectively present 37% and 41% identity with the *Anopheles gambiae*-AChE, (Figure 2). Yet despite the low identity scores, the AChE site is conserved between species, with 90% of amino acids shared. For the enzyme nAChR, *A. gossypii* and *D. melanogaster* respectively presented 29.96% and 29.39% of identity with *H. sapiens*-nAChR, with 55% of amino acids shared at the active site (Figure 3). The Cht enzyme of *A. gossypii* and *D. melanogaster* respectively presented 61% and 60% identity with *Ostrinia furnicalis*-Cht, and 100% of amino acids shared at the active site (Figure 4).

According to Cheung et al. (2018) [105], the *A. gambiae*-AChE active site amino acids are: W245, G279, S360, W441, C447, F449, E405, Y408, Y412, and H600; with 90% of these active site amino acids shared by the species under study. Luo et al. (2009) [106] has reported that the amino acids of the *H. sapiens*-nAChR active site are: Q66, C200, M125, Y197, V157, W156, Y64, Y204, and I127, with only 55% of these active site amino acids being shared by *A. gossypii* and *D. melanogaster.* However, all of the active *O. furnicalis*-Cht amino acids were highly conserved in the species under study. The Cht amino acids of *O. furnicalis* are: Y1624, W1621, N1692, W1691, E1733, and W1809 [107].

### 2.2. Homology Modeling

**Figure 5:**
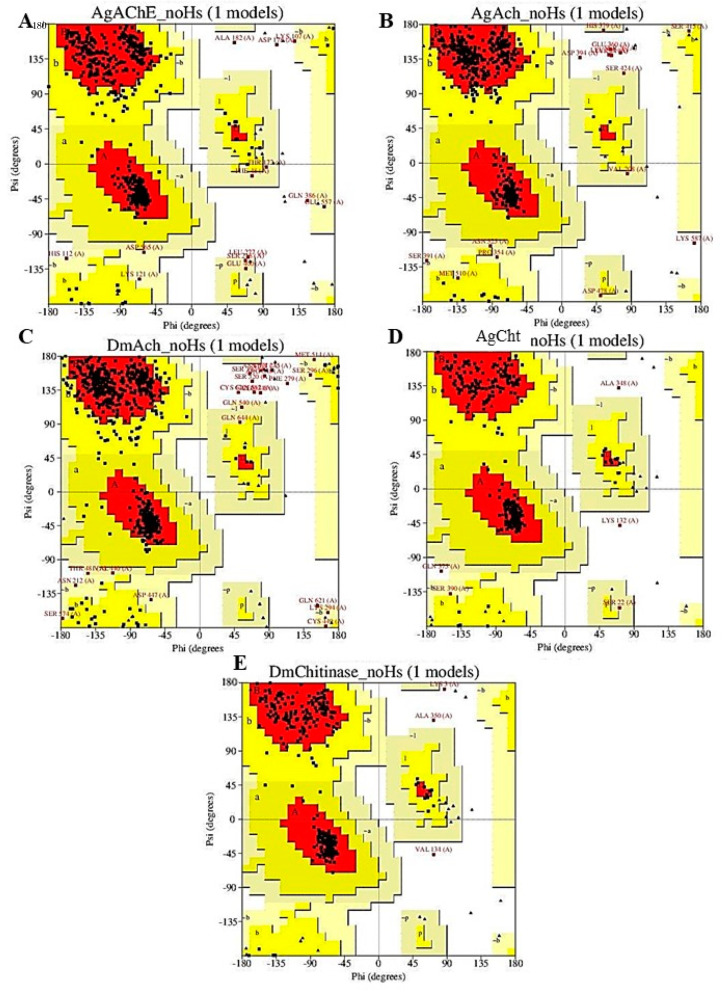
Ramachandran graphs of the homology models generated for *A. gossypii* and *D. melanogaster* enzymes. The colored regions represent the permitted and favored regions of the secondary structures and the white regions represent the prohibited regions. (**A**) *A. Gossypii*-AChE, (**B**) *A. gossypii*-nAChR, (**C**) *D. melanogaster*-nAChR, (**D**) *A. gossypii*-Cht (**E**), and *D. melanogaster*-Cht.

In this study, one AChE model, two nAChR models, and two Cht models were generated. The reliability of the models was assessed using a Ramachandran chart, which represents all possible combinations of dihedral angles Ψ (psi) versus φ (phi) for each amino acid in a protein, except glycine, which has no side chains. The model is considered to be reliable when more than 90% of the amino acids are present in the permitted and/or favored regions (colored regions of the graph). Blank regions represent outliers with poor contacts. All of the generated models presented more than 97% of their amino acids in allowed and favored regions (Figure 5 and Table 1), and were used in the following methodologies.

### 2.3. QSAR Modeling

**Figure 6:**
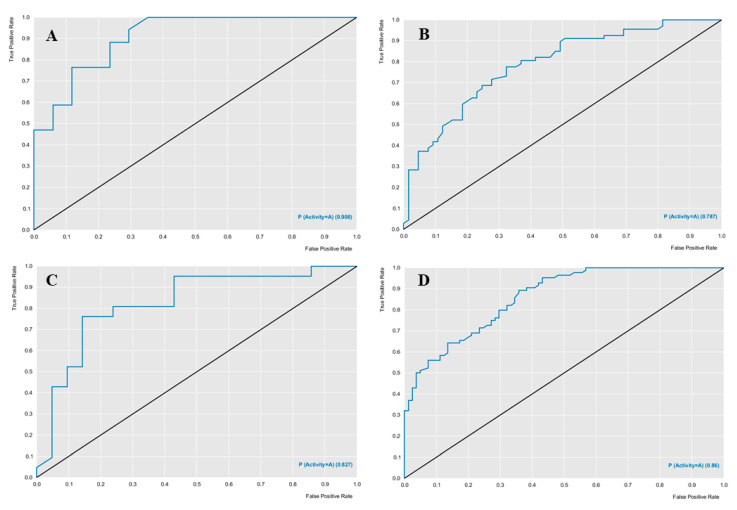
ROC curves generated from the RF models, for each species studied. ROC curve of Aphis gossipy Test (**A**) and Cross (**B**), and Drosophila mel-anogaster Test (**C**) and Cross (**D**).

The models used the RF algorithm, were built using the cross-validation procedure in the Knime software, and were evaluated for their predictive power parameters of specificity, sensitivity, accuracy, precision, (positive predicted value-PPV), and negative predicted value (NPV). Performance and robustness were evaluated using the ROC curve and the Mathews correlation coefficient (MCC). Table 2 describes the characteristics of the models in terms of predictive power and robustness, and Figure 6 presents the performance of each model. Both models presented predictive power above 70%.

The ROC curve analysis provided good results, the *Aphis gossypii* ROC curve presented a value of 0.78 and the *D. melanogaster* a value of 0.86 for cross-validation (Figure 6), both with an accuracy value higher than 70%, revealing a model with excellent classification and performance (Table 2 and Table 3). Using these models with excellent performance, diterpene and natural weed product banks were screened to select compounds potentially active against AChE, nAChR, and Cht in the studied species.

The descriptors used to generate the predictive models belong to the Dragon software, we selected the 15 most influential descriptors for the *Drosophila melanogaster* model, eight of which belong to the block of GETAWAY (GEometria, Topologia e Atom-Weights AssemblY) descriptors (ISH, H3u, HIC, HGM, H1u, H2u, HATS2u and HATS4u), these descriptors are calculated based on the representation of the molecular structure, in the Molecular Influence Matrix (MIM), denoted by H, including the atomic coordinates that are considered concerning the geometric center of the molecule, to obtain translation invariance. The other seven descriptors selected for the *Drosophila melanogaster* model belong to the block of WHIM (Weighted Holistic Invariant Molecular descriptors) descriptors (Dv, De, Vm, Vv, Ve, Vp and Vs). These descriptors are geometrical based on statistical indices calculated on the projections of the atoms along principal axes, are built in such a way as to capture relevant molecular 3D information regarding molecular size, shape, symmetry, and atom distribution concerning invariant reference frames.

As we selected the 15 most influential descriptors for the model against *Aphis gossypii* specie, five of these belong to the block GETAWAY (GEometria, Topologia e Atom-Weights AssemblY) descriptors (R2u, R3u, H5e, H6e and H8e), these descriptors use the molecular information matrix and shows rotational invariance concerning the coordinates of the molecule, thus resulting independently of the alignment of the molecule. The other ten most influential descriptors in the *Aphis gossypii* model belong to the RDF (*Radial Distribution Function*) descriptors (RDF105v, RDF110v, RDF115v, RDF120v, RDF125v, RDF130v, RDF135v, RDF140v, RDF145v and RDF150v). These descriptors are based on a radial distribution function that can be understood as the probability distribution of finding an atom in a spherical volume of radius R, taking into account the characteristics of the atoms, the interatomic distance, and the number of atoms in the molecule.

### 2.4. Combined Ligand-Based and Structure-Based Analysis

The molecular docking study was performed for the AChE, nAChR, and Cht enzymes of the species selected in this study. The diterpene bank was evaluated to select molecules with good probabilities of potential inactivation. The Molegro software generates compound interaction energies, producing a Moldock Score for each protein studied. Calculations were performed to identify the compounds presenting a higher probability of being potentially active for each protein analyzed, using the following formula:

(1) $$\mathrm{Prob}=\;\frac{E_{\mathrm{Lig}}}{E_{\mathrm{MLig}}},{\;\mathrm{se}\;E}_{\mathrm{Lig}}\;<{\;E}_{\mathrm{Inib}}$$

where E_Lig_ is the energy of the analyzed ligand, E_MLig_ is the lowest energy obtained from the tested ligands, and E_Inib_ is the energy of the PDB inhibitor ligand obtained from the crystallography data of the tested protein. Only molecules that obtained a binding energy below the binding energy of the crystallographic inhibitor ligand were considered potentially active.

Of the 1955 compounds analyzed using molecular coupling, 1702 were considered to be potentially active against *Aphis gossypii*-AChE, 1532 active against *Aphis gossypii* nAChR, 33 active against *Aphis gossypii*-Cht, 1719 active against *D. melanogaster*-AChE, 1207 active against *D. melanogaster* nAChR and 20 actives against *D. melanogaster*-Cht. Most of compounds were shown to be potentially active in both species for the enzymes AChE and ACh.

A second consensus analysis was carried out to identify potentially multi-targeting compounds, which, based on the RF model and docking, demonstrate potential active probabilities for more than one species. The following formula was used:

(2) $${\mathrm{Prob}}_{\mathrm{Comb}}\;=\;\frac{\left(\mathrm{ProbDc}\;+\;\left(1\;+\;\mathrm{ESP}\right)\;\times {\;P}_{\mathrm{Activity}}\right)}{2\;+\;\mathrm{ESP}},{\;\mathrm{Se}\;\mathrm{Prob}}_{\mathrm{Comb}}>\;0.5$$

where Prob_Dc_ is the active potential probability of the molecular coupling analysis, ESP is the specific mean value of the RF model and P_Activity_ is the active potential probability value of the RF model. This combined probability was conditioned, as only molecules with values above 0.5 were considered likely to be active. The combined probability values were calculated for the compounds identified for each target enzyme, and we analyzed which molecules were multi-targeting.

Of the 1955 compounds analyzed for combined probability (Prob_Comb_), 313 were considered potentially active against *Aphis gossyppii*-AchE, with a probability ranging from 59 to 75%, 95 were considered potentially active against *Aphis gossyppii*-nAChR with a probability ranging from 63 to 76%, 33 were considered potentially active against *Aphis gossypii*-Cht, with a probability ranging from 62 to 78%, 321 were considered potentially active against *D. melanogaster*-AchE, with a probability ranging from 54 to 75%, 74 were considered potentially active against *D. melanogaster*-nAChR, with a probability ranging from 55 to 74% and 5 were considered potentially active against *D. melanogaster*-Cht, with a probability ranging from 50 to 62%.

After performing the combined analysis, based on the ligand and structure, and using the formula to identify multitarget molecules, we identified 15 potentially active molecules for the three *A. gossypii* enzymes: AChE, nAChR, and Cht (Table 4) and 37 potentially active molecules for *D. melanogaster* enzymes: AChE and nAChR (Table 5).

The results of the ligand efficiency (LE) [108,109] are also available as a parameter to evaluate the best diterpenes (Table 4 and Table 5). The selected diterpenes for *Aphis* showed LE close to the insecticides while for *Drosophila* are similar or superior, suggesting this class of compounds as potential bioinsecticides. Additionally, flexible docking using the GOLD 5.6.2 program was performed to compare the results, being similar to the results obtained with Molegro, excepted partially for nAChR. Therefore, the results reinforce the diterpenes were selected using Molegro software (Table 4 and Table 5).

Analyzing Table 4, we observe five diterpenes that presented the highest in silico activities against *A. gossypii*. bistenuifolin L (**1836**) is an *ent*-kaurane diterpene that occurs in a species of the genus *Isodon* of the subfamily Nepetoideae (Lamiaceae), and with botanical occurrence in the region of China. In its structure, we verified the presence of a heterodimeric, a six acetoxy and two hydroxyls. The second-ranking molecule is also a *ent*-kaurane diterpene, bistenuifolin K (**1931**), which has a bicyclic structure, differing from the bistenuifolin C (**1934**) in the number of acetoxy and hydroxyl groups, presenting three acetoxy and five hydroxyl groups, while bistenuifolin C has four acetoxy and hydroxyl groups. Bistenuifolin I (**1800**), also an *ent*-kaurane diterpene with four hydroxyls, acetoxy and carbonyl groups, occurs botanically in *Isodon* species, although its geographical location has China. The fifth molecule with high activity is inermes b (**1936**), a *neo*-clerodane diterpene, which has another presents subtype of diterpene and diverges from **1931** and **1934**. Like some other diterpenes already mentioned, inermes b can be found in China, in species of the genus *Isodon*.

In Table 5, analyzing the five diterpenes that presented potential in silico activity against *D. melanogaster* one observes that the clerodane diterpene salvisplendin C (**1086**) is found mainly in the genus *Salvia,* subfamily Nepetoideae (Lamiaceae) and distributed in Italy (Table 6). Diterpene **1086** has one hydroxyl and a lactonic ring in its structure, providing the highest activity of *D. melanogaster*. The with a carbocyclic skeleton diterpene salvixalapadiene (**1195**) has two carbonyls and two lactones in its chemical structure, present in Lamiaceae in the genus *Salvia* and geographically in Mexico. The *ent*-kaurane diterpene racemosin A (**1302**), obtained from the leaves of the species *Isodon henryi*, also has two acetoxy, a carbonyl and a hydroxyl group. Salviarin (**1027**) is also a clerodane diterpene, however, the substituents differ considerably when compared to the diterpene classified as the most active (**1086**), considering that **1027** has one hydroxyl groups and two carbonyls: occurring in six species of the subfamily Nepetoideae. The seco-neoclerodane diterpene tonalensin (**342**), less active compared to the two most active diterpenes (clerodanes), has functional groups ether, carbonyl, and lactone, and can be found in Mexico in species of the genus *Salvia*.

Information on the distribution of diterpenes considered active against *A. gossypii* and *D. melanogaster* is contained in Table 6 and Table 7.

### 2.5. Toxicity

Toxicity was evaluated *A. gossypii* and of the 15 compounds considered potentially active in the RF model and docking, 11 compounds **1800**, **1804**, **1836**, **1840**, **1842**, **1845**, **1910**, and **1931**–**1934** presented no predicted mutagenicity or tumorigenesis effect, or negative effects on the reproductive system, or irritability. These molecules were considered to possess the best properties for not presenting any toxicity risk. Table 8 presents the compounds with no toxicity for the evaluated parameters.

Toxicity was also evaluated for *D. melanogaster*, and of the 37 compounds considered potentially active in the RF model and docking, 27 compounds **21**, **44**, **46**, **47**, **51**, **67**, **77**, **95**, **111**, **131**, **151**, **199**, **200**, **231**, **342**, **434**, **442**, **483**, **759**, **787**, **1015**, **1027**, **1086**, **1184**, **1195**, **1302**, and **1350** presented no predicted mutagenicity or tumorigenesis effect, or negative effects on the reproductive system, or irritability. These molecules were considered to possess the best properties; for not presenting any toxicity risk. Table 9 presents the compounds with no toxicity for the evaluated parameters.

### 2.6. Interaction Analysis

Of the compounds considered potentially active in the RF model, in docking, and multitarget, with higher LE values and with low toxicity for the species under study, we selected the two compounds with the highest Prob_Comb_ value to analyze interactions.

#### 2.6.1. Acetylcholinesterase

**Figure 7:**
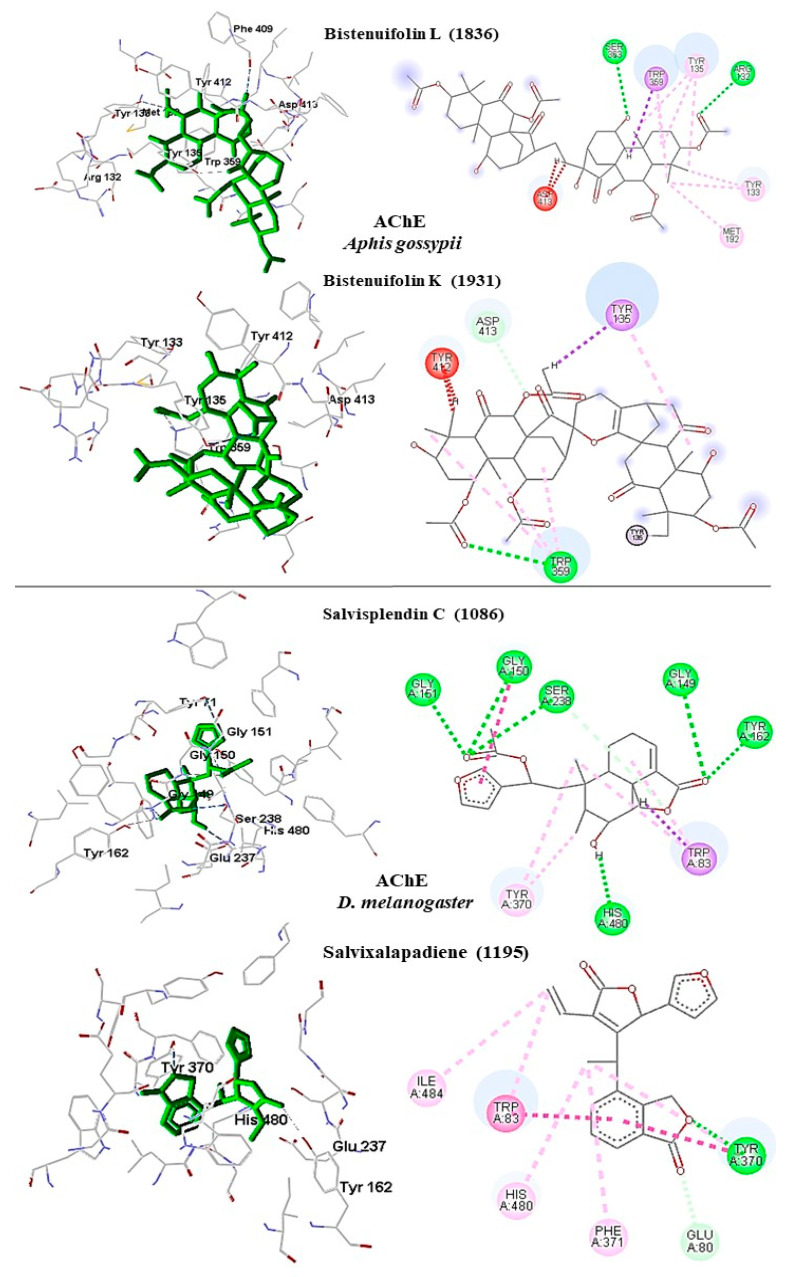
Interactions (3D and 2D) between **1836** and **1931** and *A. gossypii*-AchE; and between **1086** and **1195** and *D. melanogaster*-AChE. Hydrogen bonds are highlighted in green, hydrophobic interactions are highlighted in pink, and steric interactions are highlighted in red.

**Figure 8:**
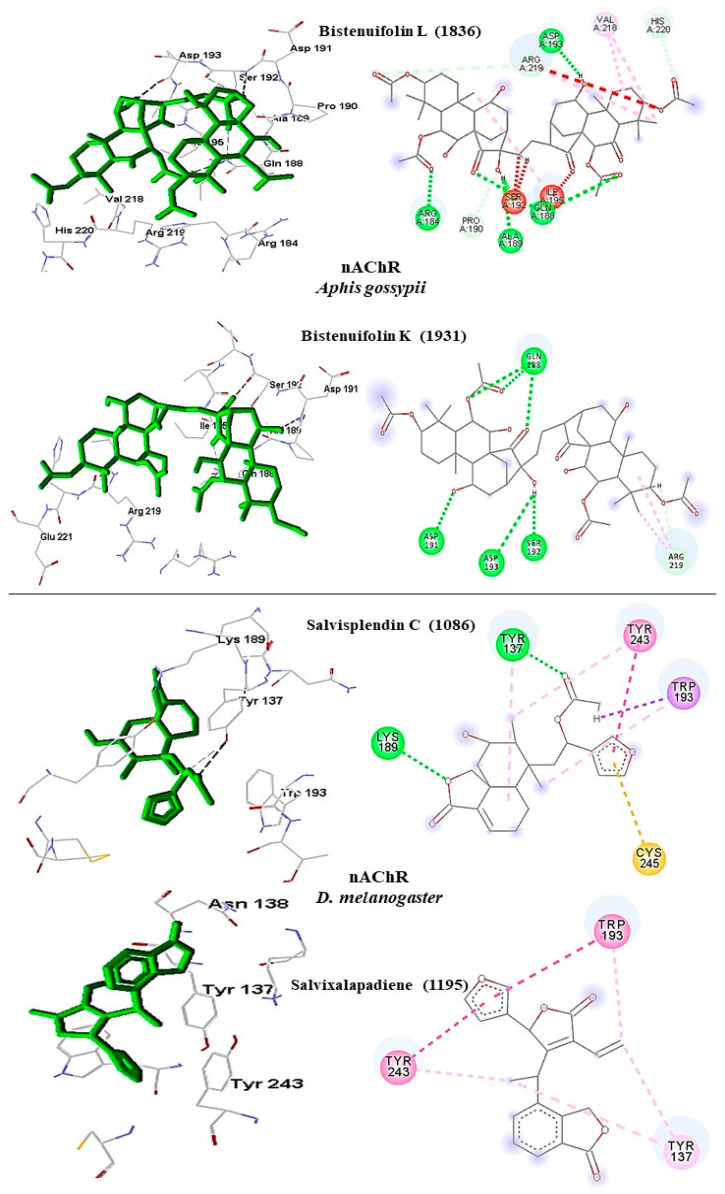
Interactions (3D and 2D) between **1836** and **1931** and *A. gossypii*-nAChR; and between **1086** and **1195** and *D. melanogaster*-nAChR. Hydrogen bonds are highlighted in green, hydrophobic interactions are highlighted in pink, and steric interactions are highlighted in red.

In *A. gossypii*-AchE, the diterpene bistenuifolin L (**1836**) formed four hydrogen bonds with the amino acids Tyr135, Trp359, Leu366, and Val415. In addition, it formed thirteen hydrophobic interactions, especially Tyr133 and Phe409, with interaction values equal to −18.56 and −17.31 kcal mol^−1^ respectively (Table 10) [111]. The bistenuifolin K (**1931**) compound formed a stable bond with Tyr135 with an interaction value of −18.49 kcal mol^−1^ and several hydrophobic interactions with Tyr133, Glu131, Trp359, Phe368, Lys356, Phe409, and Asp413 (Figure 7). The interaction values ranged from −2 to −43 kcal mol^−1^. The insecticide Chlorpyrifos did not show any hydrogen bond but five hydrophobic interactions with the amino acids; Tyr133, Tyr135, Trp359, Tyr412, and Asp413 (Figure 8). The interaction values ranged from −1 to −24 kcal mol^−1^.

In addition, in *D. melanogaster*-ChE, the compound salvisplendin C (**1086**) formed several hydrogen bonds with the amino acids Tyr71, Gly149, Gly151, Tyr162, Ser238, and His480. It also formed two hydrophobic bonds with the amino acids Tyr71 and Glu237 (Figure 7). It was observed that the energetic contributions of the interactions varied from −4 to −11 kcal mol^−1^ (Table 11) [111]. The compound salvixalapadiene (**1195**) has less-stable interactions; with only one hydrogen bond with the Tyr370 residue with an interaction value of −29.72 kcal mol^−1^. Also, it shows two hydrophobic interactions with the amino acids Glu237 and Hist480. The diterpenes have stronger interactions than the insecticide Methomyl, which formed only one hydrophobic bond with His480 (−5.26 kcal mol^−1^) and another with Trp83 (−26.29 kcal mol^−1^) (Figure 8).

#### 2.6.2. Nicotinic Acetylcholine Receptor

**Figure 9:**
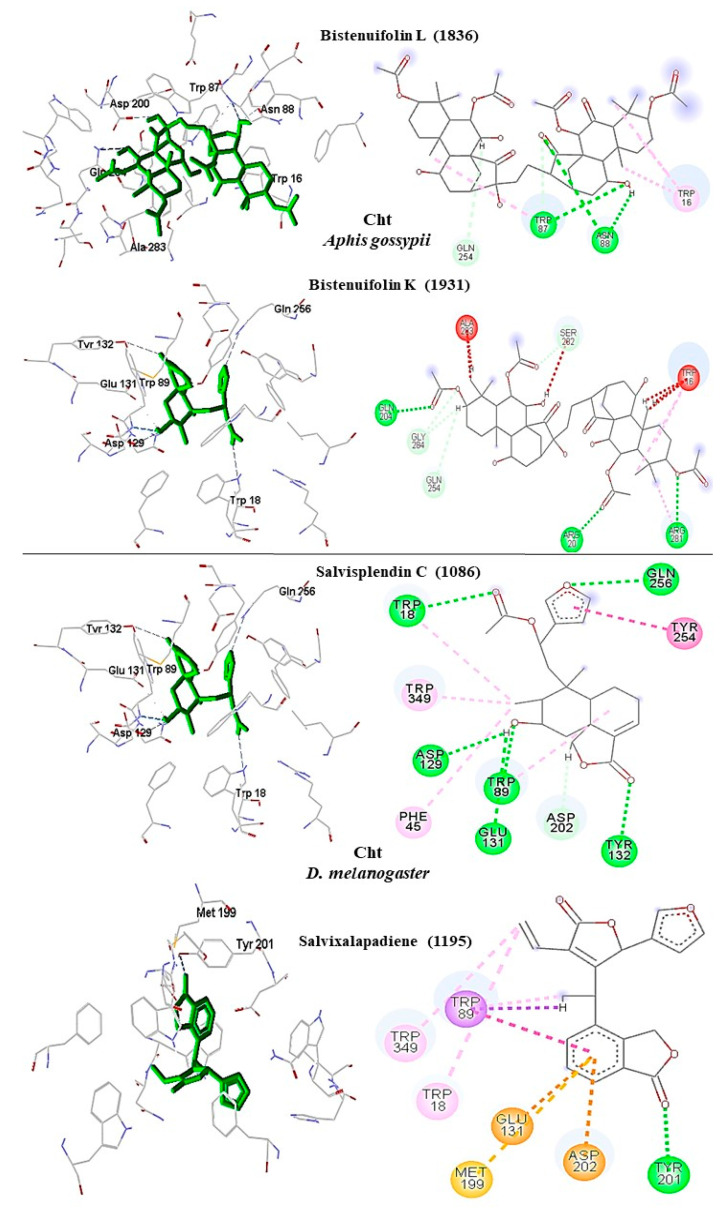
Interactions (3D and 2D) between **1836** and **1931** and *A. gossypii*-Cht; and between **1086** and **1195** and *D. melanogaster*-Cht. Hydrogen bonds are highlighted in green, hydrophobic interactions are highlighted in pink, and steric interactions are highlighted in red.

**Figure 10:**
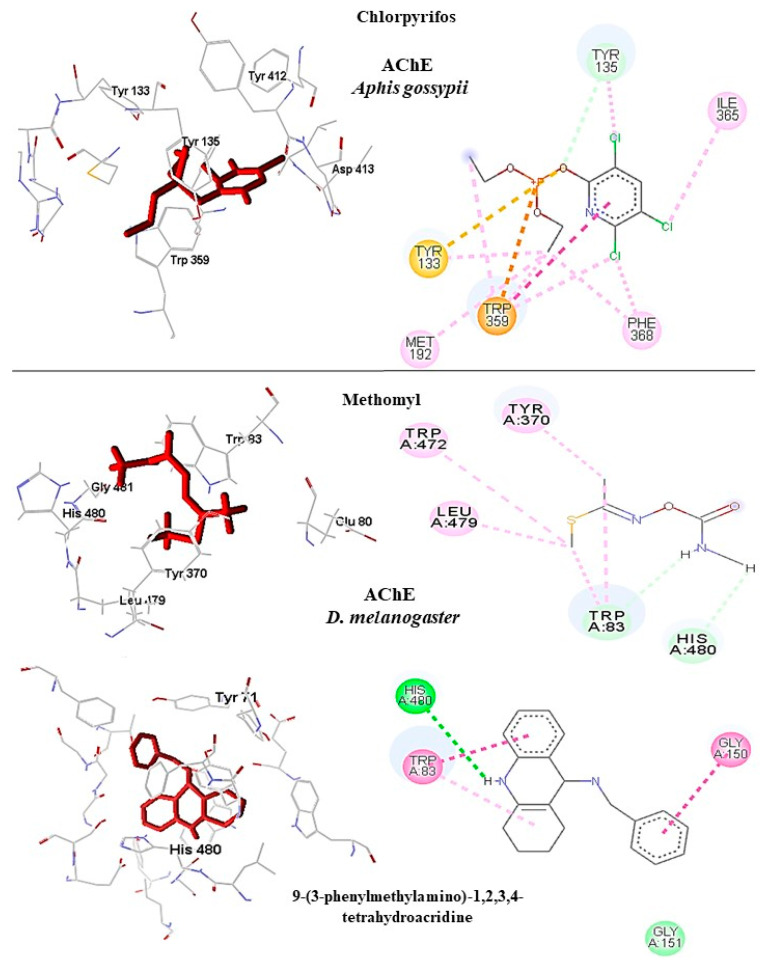
Interactions (3D and 2D) between Chlorpyrifos and *A. gossypii*-AchE; between both Methomyl and *D. melanogaster*-AchE, and 9-(3-phenylmethylamine)-1,2,3,4-tetrahydroacridine and *D. melanogaster*-AchE. Hydrogen bonds are highlighted in green, hydrophobic interactions are highlighted in pink, and steric interactions are highlighted in red.

In *A. gossypii*-AChE, bistenuifolin L (**1836**) presented four important hydrogen bonds, with Ala189, Pro190, Asp191, Ser192, and nine hydrophobic interactions with the residues Gln188, Ala189, Pro190, Asp191, Ser192, Ile195, Asp193, Arg220, and His220 (Figure 9). Among these amino acids, Asp191 stands out, with an interaction value of 19.66 kcal mol^−1^. The bistenuifolin K compound (**1931**) showed only one hydrogen bond with the residue Ser192, but it presented several hydrophobic interactions with the amino acids Gln188, Ala189, Pro190, Asp191, Ser192, Ile195, Val218, Arg219, and His220. Among the diterpenes, the bistenuifolin L compound showed a higher interaction value, especially Pro191 (−29.03 kcal mol^−1^) (Table 10) [111]. The insecticide Clothianidin formed three hydrogen bonds; with the amino acids Ala189, Ala189, Ser192, and two hydrophobic interactions with Ala189 and Ser192 (Figure 10).

In *D. melanogaster*, salvisplendin C (**1086**) formed two hydrogen bonds with the amino acids Tyr137 and Lys189 and two hydrophobic interactions with the amino acids Tyr137 and Trp193 (Figure 9). Among these amino acids, Tyr137 stands out, with an interaction value with the diterpene corresponding to −28.27 kcal mol^−1^ (Table 11) [111]. The compound salvixalapadiene (**1195**) showed only hydrophobic interactions with the Tyr137 and Tyr243 residues. Already the insecticide acetamiprid formed only with a hydrogen bond with the amino acid Tyr137 and hydrophobic interaction with the amino acid Tyr243 (Figure 10).

#### 2.6.3. Chitinase

The Chitinase enzyme presents over 2000 amino acids in its protein structure, yet the N-terminal region alone is responsible for its activity. Thus, homology models were built with this region only, and the amino acids in the docking images were renamed. The correct (non-altered) numbering of the amino acids corresponding to the N-terminal region of Cht is indicated in parentheses.

**Figure 11:**
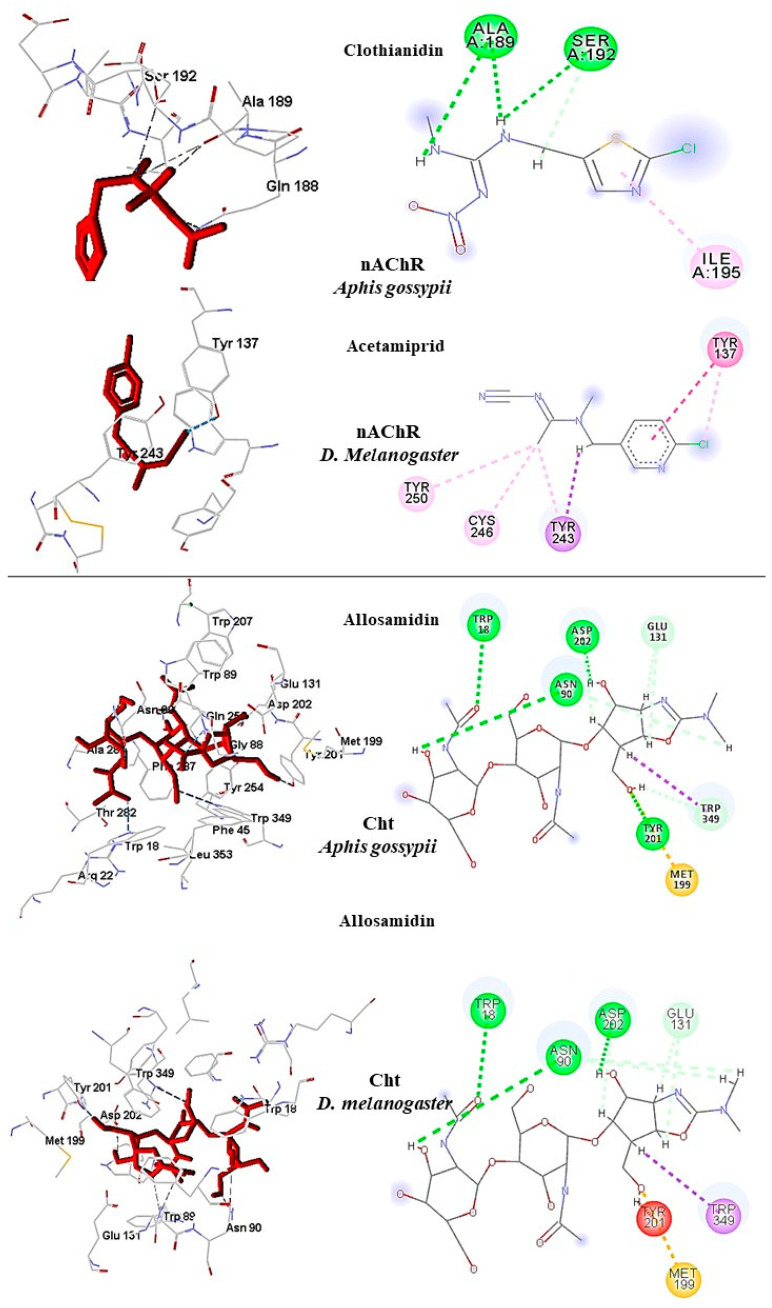
Interactions (3D and 2D) between clothianidin and *A. gossypii*-nAChR; Acetamiprid and *D. melanogaster*-AchE; Allosamidin and *A. gossypii*-Cht; and Allosamidin and *D. melanogaster*-Cht. Hydrogen bonds are highlighted in green, hydrophobic interactions are highlighted in pink, and steric interactions are highlighted in red.

In *A. gossypii* Cht, the bistenuifolin L (**1836**) compound had four hydrogen bonds; with the amino acids of the active site Trp16 (W1355), Glu204 (Q1543), Arg281 (R1620), and Ser282 (S1621). Also, it formed eleven hydrophobic interactions with the amino acids Trp16 (W1355), Tyr19 (Y1358), Trp87 (W1426), Asn88 (N1427), Phe285 (F1624), Glu204 (Q1543), and Ser282 (S1621), among others (Figure 11). Among these amino acids, Trp87 (W1426) stands out with an interaction value of −32.65 kcal mol^−1^ (Table 10) [111]. The compound salvixalapadiene (**1195**) showed six hydrogen bonds with the amino acids Trp16 (W1355), Arg20 (R1359), Tyr130, Gln254 (Q1593), Tyr199 (Y1538), and Trp347 (W1686). Among these amino acids, Trp16 (W1355) stands out with an interaction value of −30.84 kcal mol^−1^ (Table 10). In addition, fourteen hydrophobic interactions were observed. Due to a large number of hydrogen bonds, these complexes are considered stable. The interactions of the insecticide Allosamidin with the active site Cht also proved to be stable, presenting through six hydrogen bonds with the amino acids Trp16 (W1355), Arg20 (R1359), Trp87 (W1426), Asn88 (N1427), Asp200 (D1539), and Gln254 (Q1593) (Figure 10).

In *D. melanogaster* Cht, the compound salvisplendin C (**1086**) showed hydrogen bonds with the amino acids Trp18, Trp89 (W1045), Trp129 (W1085), Tyr132 (Y1088), Trp349 (W1305), and Gln256 (Q1212) and hydrophobic interaction with the Glu131 residue (E1087). Among these interactions, Trp89 (W1045) with interaction value −28.39 kcal mol^−1^ (Table 11) stands out [111]. Salvixalapadiene (**1195**) showed only one hydrogen bond with the amino acid Tyr201 (Y1157) and two hydrophobic interactions with residues Met199 (M1155) and Tyr201 (Y1157) (Figure 11).

The insecticide Allosamidin formed four hydrogen bonds with amino acids Trp18 (W974), Asn90 (N1046), Tyr201 (Y1157), Asp202 (D1158), and a steric bond with amino acid Trp89 (W1045). The insecticide interactions observed in this study are presented in Figure 10.

## 3. Material and Methods

### 3.1. Alignment of Protein Sequences

The AChE, nAChR, and Cht enzyme sequences from *A. gossypii* and *D. melanogaster* were obtained from GenBank [112], and global alignment was performed using the Clustal Omega web tool [113], which aligns protein sequences inserted by the user. Unshared end regions were excluded from the alignment. The alignment facilitated the investigation of active sites, determination of similarities, and shared identity between the enzymes in the two species under study.

### 3.2. Homology Modeling

Due to the lack of experimentally known 3D protein structures, homology models of the enzymes under study for *Aphis gossypii* and *Drosophila melanogaster* were built. The sequences of the enzymes and species selected in the study were obtained from the GenBank database [114], and the model structures were obtained from the Protein Data Bank (PDB) [115]. Three enzymes were selected for the construction of homology models: AChE, ACh, and Cht. The template enzymes were: AChE from *Anopheles gambiae* (PDB ID: 5YDH) [116], ACh from *Homo sapiens* (PDB ID: 6PV7) [117], and ChtII from *Ostrinia furnicalis* (PDB ID: 6JAV) [107]. The enzyme models were built using the molecular homology modeling method in the MODELLER 9.20 software [118]. Five models were generated and the lowest energy model was chosen. The model’s stereo-chemical qualities were evaluated using the PSVS web server (protein structure validation software suite) (http://psvs-1_5-dev.nesg.org/), and PROCHECK [119]. PROCHECK generates a Ramachandran graph [120], which determines allowed and disallowed regions of the main chain of amino acids.

### 3.3. Molecular Docking

Molecular docking calculations flexible approach were carried out by the *Molegro Virtual Docker (MVD) 6.0* software [121], and three targets were selected for anchorage studies. The 3D structure of the *Drosophila melanogaster*-AChE enzyme was obtained from the Protein Data Bank (PDB), using the following code: PDB ID 1DX4 [122]. *Aphis gossypii*-AChE, *A. gossypii*-nAChR and *D. melanogaster*-nAChR, and *A. gossypii*-Cht and *D. melanogaster*-Cht were obtained by homology. Initially, all water molecules were removed from the crystalline structure and the mean square quadratic deviation (RMSD) was calculated from the poses, indicating the degree of reliability of the adjustment. The RMSD provides the connection mode close to the experimental structure and is considered successful if the value is below 2.0 Å. The MolDock score was used as a scoring function to predict the best interactions between the ligand and the receptor. The anchor assistant was then generated, and the enzyme and ligands were inserted to analyze the stability of the system based on the interactions identified with the active site of the enzyme.

Enzyme information is contained in Table 12. In addition, we consider the connection ligand efficiency values (LE).

The program GOLD 5.6.2 [123] was used to perform flexible docking with the compounds selected using Molegro software. The parameters are standardized by the program, which makes the random search of the conformational space, decreasing the probability of the ligand being stuck in minimal locations. The scoring function selected was GoldScore. This function is similar to molecular mechanics with four terms:

Goldsore = Shb_ext + Svdw_ext + Shb_int + Svdw_int(3)

where, Shb_ext is the hydrogen protein ligand binding score, Svdw_ext is the van der Waals protein ligand score, Shb_int is the contribution of the ligand’s intramolecular and hydrogen bonds; and Svdw_int is the contribution of intramolecular agglutinating stress [123].

#### 3.3.1. Docking Parameters

##### Molegro Virtual Docker (MVD) 6.0

Molecular docking calculations were performed using Molegro Virtual Docker (MVD) 6.0 [121]. A template docking was created on the enzyme using the as ligands, the respective insecticides of the enzymes under study (Table 12). Next, a docking wizard was created, in which the enzyme molecules and ligands were inserted, to analyze the stability of the system through the interactions identified with the active site of the enzyme, taking as reference the energy value of the MolDock Score. The molecular docking calculations were performed in MVD. Different search functions were analyzed and some parameters such as minimization steps per residue and per run, number of runs and iterations, and population size, were evaluated and defined according to their capacity to reproduce the crystal structure of the complex [121]. The algorithm MolDock SE (Simplex Evolution) was used with the following parameters: A total of 10 runs with a maximum of 1500 iterations using a population of 50 individuals, 2000 minimization steps for each flexible residue, and 2000 steps of global minimization per run. The MolDock Score (GRID) scoring function was used for calculating the docking energy values. A GRID was set at 0.3 A and the search sphere was fixed at 15 A of radius. For the ligand energy analysis, the internal electrostatic interactions, internal hydrogen bonds, and sp2-sp2 torsions were evaluated. To validate the docking methodology, we carried out the insecticide binding method in the enzyme structures with an average square distance value (RMSD) with values below 2.0 Å (Table 12) [121].

##### GOLD 5.6.2

GOLD gives the best poses by a genetic algorithm (GA) search strategy, and then various molecular features are encoded as a chromosome [124]. GOLD uses a genetic algorithm (GA) in which the following parameters are optimized: Dihedrals of ligand rotatable bonds; ligand ring geometries; dihedrals of protein OH groups and NH3 groups; and the mappings of the fitting points [123]. Initially, the protein is imported and the water molecules and cofactors are removed. Then hydrogens are added throughout the protein. We selected the option to detect the cavity of the active site using the template at a distance of 10 Angstrons. Then the compounds in sdf are imported and we chose the GoldScore scoring function. The GoldScore was used as an empirical scoring function because it has been shown to outperform the other GOLD scoring schemes [125]. The default calculation form, which provides the most accurate docking results, was selected for all calculations. In the standard calculation mode, by default, the GA run comprised 100 000 genetic operations on an initial population of 100 members divided into five subpopulations, and the annealing parameters of fitness function were set at 4.0 for van der Waals and 2.5 for hydrogen bonding [123,125].

### 3.4. Data Collection and Handling

Chemical compounds with known activity against the following species or genus were selected: *Aphis* (*Aphis gossypii* CHEMBL613807, *Aphis fabae* CHEMBL2366919, *Aphis craccivora* CHEMBL613806, *Aphis medicaginis* CHEMBL2367276), and *Drosophila melanogaster* (CHEMBL2366447, CHEMBL2366447, CHEMBL2366467, CHEMBL2366467, CHEMBL2366467, CHEMBL2366467, CHEMBL2366467, and CHEMBL2366470). These compounds from the CHEMBL database were used to build predictive models [126]. The details can be found in Table 13.

The compounds were classified as active or inactive according to the type of biological activity [127,128,129]. A database comprised of 1955 diterpenes isolated from species of the Nepetoideae subfamily (Lamiaceae) was also used; obtained in SistematX (https://sistematx.ufpb.br) [110]. All prediction diterpenes were evaluated by virtual screening to identify molecules with potential activity against AChE in each species, in accordance with workflows presented by Fourches et al. [130,131,132]. The three-dimensional structures were generated using the Chemaxon Standardiser v.18.17.0 (www.chemaxon.org) [133].

### 3.5. Quantitative Structure-Activity Relationship (QSAR) Modeling

Knime 3.5.3 software (KNIME 3.5.3, Konstanz Information Miner Copyright, 2018, www.knime.org) [134] was used to perform analyses and generate in silico models. Given the success of our previous studies [52,83,122,135,136,137], we chose to perform a 3D QSAR analysis for each species or genus bank. All compounds studied with a resolved chemical structure were saved in the special data file format (SDF), and imported into the Dragon 7.0 software [138], to generate descriptors.

The banks of molecules and their calculated descriptors were imported from the Dragon software, the data was divided as a “Partitioning” tool, using the “Stratified sample” option, which separated the data into training and test sets, respectively representing 80% and 20 % of the compounds. The sets were randomly selected, yet the proportions of active and inactive substances were maintained in both databases.

The Random Forest (RF) algorithm, using WEKA nodes [139] was used to build predictive models. The parameters selected for the RF algorithm were as follows for all models: Total number of forests = 250, and one seed was used to generate random numbers. Cross-validation was performed to estimate the predictive power of the models developed.

The external performances of the selected models were analyzed for sensitivity (true-positive rate or active rate), specificity (true-negative rate or inactive rate), and accuracy (general predictability). The sensitivity and specificity of the receiver’s operating character curve (ROC) was used since it describes the actual performance more clearly than does accuracy-general predictability.

The models were also analyzed using the Matthews correlation coefficient (MCC), which can evaluate the model globally based on the results obtained in the confusion matrix. The MCC is a correlation coefficient for the observed and predictive binary classifications, resulting in values of between −1 and +1, where a coefficient of +1 represents a perfect forecast, 0 represents a random forecast and −1 indicates total disagreement between prediction and observation [140].

The MCC can be calculated using the following formula:

(4) $$\mathrm{MCC}=\;\frac{\mathrm{VP}\;\times \;\mathrm{VN}\;-\;\mathrm{FP}\;\times \;\mathrm{FN}}{\surd \left(\mathrm{VP}+\mathrm{FP}\right)\left(\mathrm{VP}+\mathrm{FN}\right)\left(\mathrm{VN}+\mathrm{FP}\right)\left(\mathrm{VN}+\mathrm{FN}\right)}$$

where VP represents true positives, VN represents true negatives, FP represents false positives, and FN represents false negatives.

The applicability domain (APD) was used to analyze the compounds in the test sets to assess whether the predictions were reliable. The APD is a theoretical chemical space that involves the model descriptors and the modeled response, estimating uncertainty when predicting a compound’s activity in the training set used in the development of the model. This technique is important for verifying the reliability of QSAR models, comparing predicted values with observed values [141].

The APD is calculated using the following formula:

APD = d + Zσ(5)

where d and σ are respectively the Euclidean distances and the mean standard deviation for the compounds in the training set. Z is an empirical cutoff value, which in this study was set to 0.5 [142].

### 3.6. Toxicity

OSIRIS Property Explorer (https://www.organic-chemistry.org/prog/peo/) [143] generally allows designing chemical structures and predicting ADMET profiles [144]. In this study, we used OSIRIS to analyze toxicity. Four predictive parameters were provided by the software: Mutagenicity, tumorigenicity, reproductive effect, and irritability.

## 4. Conclusions

The insect species studied in this work, *Aphis gossypii* and *Drosophila melanogaster*, cause damages to both agriculture and man. In addition, improper use of insecticides to combat these and other pests, has resulted in accumulation of toxic effects in beneficial organisms as well, this, while harming ecosystems due to soil contamination. Through varied computational approaches, the present study aimed to identify potential bioinsecticides with low toxicity.

Bioinsecticide designs against essential targets, in five homology models, were built for enzymes not available in databases. The selected enzymes were AChE, nAChR, and Cht. On the Ramachandran graph, the models presented more than 96% reliability.

Predictive models were also built to predict the biological activity of diterpenes against *A. gossypii* and *D. melanogaster*. In this study, the models obtained excellent performance results, for the models, the *Aphis gossypii* ROC curve presented a value of 0.78 during cross-validation and for *D. melanogaster* a value of 0.86, both with an accuracy greater than 70%. To increase the predictive power and decrease the number of false positives generated by these models, combined analysis, based on the ligand and structure, was used. The combined analysis, based on the Random Forest and docking models, was able to identify potentially active molecules.

In this work, we apply methodologies to develop predictive QSAR models using Random Forest, homology models, molecular docking, combined ligand, and structure-based analysis, and toxicity to verify the interaction of important enzymes involved in the mechanisms of action of commercial insecticides for *Aphis gossypii* species and *Drosophila melanogaster* and evaluate the performance of 1955 diterpenes (Lamiaceae). As a result of the QSAR modeling, 11 diterpenes were selected with promising potential activity against *Aphis gossypii* and 27 structures against *Drosophila melanogaster*.

Out of 1955 diterpenes that were analyzed by combined probability (Prob_Comb_), several potentially active compounds were identified: 313 were considered potentially active against (*Aphis gossypii*-AChE) with probability potentials ranging from 50 to 73%, 95 were active against (*Aphis gossypii*-AChE) with potentials between 50 to 76%, 33 were active against (*Aphis gossypii*-Cht) with potentials between 55 to 78%, 321 were active against (*D. melanogaster*-AChE) with potentials between 50 to 68%, 74 were active against (*D. melanogaster*-nAChR) with potentials between 50 to 58% and 5 were active against (*D. melanogaster*-Cht) with potentials between 50 to 62%. We also identified 15 potentially active molecules for the enzymes, AChE, nAChR, and Cht from *A. gossypii*, and 37 potentially active molecules for *D. melanogaster* for the enzymes: AChE and nAChR.

Toxicity was also evaluated for the species *A. gossypii,* and of the 15 compounds considered potentially active in the RF model and in docking, 11 compounds presented no toxicity for the evaluated parameters. The compounds were **1800**, **1804**, **1836**, **1840**, **1842**, **1845**, **1910**, and **1931**–**1934**. Among the 37 compounds considered potentially active in the RF model and in docking against *D. melanogaster*, 27 compounds presented no toxicity. The compounds were: **21**, **44**, **46**, **47**, **51**, **67**, **77**, **95**, **111**, **131**, **151**, **199**, **200**, **231**, **342**, **434**, **442**, **483**, **759**, **787**, **1015**, **1027**, **1086**, **1184**, **1195**, **1302**, and **1350**.

We also analyzed the structures of the diterpenes presenting significant results and noticed that the most active were *ent*-kaurane, kaurane, and clerodane. We also found that the interactions formed by these compounds were either more stable or similar to the commercialized insecticides. Overall, we conclude that the compounds bistenuifolin L (**1836**), and bistenuifolin K (**1931**), are potentially active against *A. gossypii* enzymes. Salvisplendin C (**1086**) and salvixalapadiene (**1195**), are potentially active against *D. melanogaster*. We further suggest that in species studied, these diterpenes: Bistenuifolin L (**1836**), bistenuifolin K (**1931**), together with salvisplendin C (**1086**), and salvixalapadiene (**1195**), deserve to be highlighted because of their high probability of activity and low toxicity.

## Figures and Tables

**Table 1:** Percentage of amino acids present in the permitted and favored regions of the Ramachandran chart for each model.

Enzyme	Species	Ramachandran Percentage
AChE	*A. gossypii*	97.5%
nAChR	*A. gossypii*	97.4%
	*D. melanogaster*	96.9%
Cht	*A. gossypii*	98.5%
	*D. melanogaster*	99.1%

**Table 2:** Performance summary presenting the results obtained for all random forest models.

Species	Validation	Accuracy	Sensitivity	Specificity	PPV	NPV	MCC
*Aphis gossypii*	Test	0.74	0.76	0.76	0.76	0.76	0.52
	Cross	0.70	0.73	0.67	0.70	0.70	0.40
*Drosophila melanogaster*	Test	0.76	0.76	0.76	0.76	0.76	0.52
	Cross	0.73	0.76	0.71	0.73	0.74	0.47

**Table 3:** Values for the ROC curves, during the test and cross-validation, for each RF model.

Enzyme	ROC Curve	
	Test	Cross
*Aphis gossypii*	0.90	0.78
*D. melanogaster*	0.82	0.86

**Table 4:** Moldock Score and GoldScore values obtained from docking and combined probability values (Prob_Comb_) between prediction models and molecular coupling analysis for potential activity against *A. gossypii*. In the table, only the Prob_Comb_ only values considered active are presented. Data on the ligand efficiency (LE) of the compounds are also available in the table.

ID	GoldScore Flexible			Moldock Score Flexible			Prob_Comb_			LE			Prob_Activity_
	AChE	nAChR	Cht	AChE	nAChR	Cht	AChE	nAChR	Cht	AChE	nAChR	Cht	
**1800**	51.28	34.20	94.04	−174.03	−148.66	−224.29	0.70	0.74	0.70	−2.63	−2.25	−3.39	0.61
**1804**	40.87	31.15	113.8	−120.62	−135.97	−219.43	0.64	0.74	0.75	−1.94	−2.19	−3.53	0.66
**1809**	35.86	21.23	170.0	−223.77	−138.22	−176.35	0.75	0.70	0.73	−4.30	−2.65	−3.39	0.61
**1836**	42.24	23.48	127.8	−217.92	−162.71	−240.23	0.76	0.76	0.76	−3.11	−2.32	−3.43	0.62
**1840**	42.40	25.73	142.0	−108.98	−111.3	−219.83	0.63	0.68	0.78	−1.72	−1.76	−3.48	0.66
**1842**	60.92	32.90	45.7	−162.09	−140.75	−181.34	0.73	0.76	0.64	−2.57	−2.23	−2.87	0.66
**1845**	44.70	32.30	120.2	−149.98	−129.73	−179.35	0.68	0.73	0.71	−2.34	−2.02	−2.80	0.65
**1854**	41.30	32.00	71.5	−162.01	−139.78	−194.60	0.64	0.70	0.62	−3.24	−2.79	−3.89	0.57
**1855**	35.71	27.91	76.3	−148.07	−107.61	−170.44	0.62	0.63	0.61	−2.84	−2.06	−3.27	0.58
**1910**	33.08	33.24	77.8	−126.98	−148.83	−198.84	0.59	0.72	0.64	−2.39	−2.80	−3.75	0.58
**1931**	45.20	36.38	99.8	−180.50	−140.90	−197.69	0.73	0.76	0.71	−2.86	−2.23	−3.13	0.66
**1932**	45.07	29.28	92.2	−149.64	−141.42	−183.22	0.68	0.74	0.68	−2.37	−2.24	−2.90	0.64
**1933**	32.84	30.35	79.4	−142.05	−147.59	−191.20	0.64	0.75	0.66	−2.15	−2.23	−2.89	0.63
**1934**	41.82	30.02	125.7	−154.26	−89.66	−195.89	0.70	0.65	0.74	−2.41	−1.40	−3.06	0.67
**1936**	28.17	32.95	63.9	−172.98	−139.01	−206.48	0.70	0.76	0.69	−2.36	−1.90	−2.82	0.67
Insecticide	29.34	39.26	62.0	−88.38	−55.89	−146.23	-	-	-	−5.19	−3.7	−3.4	-

**Table 5:** Moldock Score and GoldScore values obtained from docking and combined probability values (Prob_Comb_) between prediction models and molecular coupling analysis for potential activity against *D. melanogaster*. In the table, only the Prob_Comb_ values considered active are presented. Data on the ligand efficiency (LE) of the compounds are also available in the table.

ID	GoldScore Flexible			Moldock Score Flexible			Prob_Comb_			LE			Prob_Activity_
	AChE	nAChR	Cht	AChE	nAChR	Cht	AChE	nAChR	Cht	AChE	nAChR	Cht	
**21**	35.26	31.61	33.90	−122.70	−66.47	−88.32	0.67	0.65	-	−4.90	−2.65	−3.53	0.58
**44**	33.55	27.08	32.12	−117.86	−80.01	−106.16	0.66	0.67	-	−4.71	−3.20	−4.24	0.58
**46**	32.36	25.65	31.48	−127.86	−77.01	−90.17	0.67	0.66	-	−5.11	−3.08	−3.60	0.57
**47**	32.63	28.45	42.33	−125.16	−83.84	−107.76	0.67	0.68	-	−5.00	−3.35	−4.31	0.57
**51**	36.68	33.86	42.50	−138.59	−87.14	−92.41	0.70	0.71	-	−5.54	−3.48	−3.69	0.57
**67**	38.28	24.51	33.76	−124.82	−91.09	−99.81	0.67	0.69	-	−4.99	−3.64	−3.99	0.56
**77**	22.53	26.14	38.41	−133.84	−84.06	−109.86	0.69	0.71	-	−5.35	−3.36	−4.39	0.62
**95**	30.27	23.47	33.67	−138.23	−79.21	−116.49	0.69	0.66	-	−5.52	−3.16	−4.65	0.58
**111**	35.09	28.31	31.66	−125.26	−82.99	−100.81	0.66	0.67	-	−5.01	−3.31	−4.03	0.56
**131**	33.19	24.81	29.23	−91.223	−71.29	−99.12	0.57	0.60	-	−3.64	−2.85	−3.96	0.52
**151**	23.97	25.21	31.02	−142.60	−87.13	−93.69	0.71	0.71	-	−5.48	−3.35	−3.60	0.61
**163**	23.82	26.01	32.97	−157.95	−78.03	−114.73	0.72	0.67	-	−6.07	−3.00	−4.41	0.58
**164**	25.68	29.30	29.76	−157.91	−78.19	−114.72	0.72	0.68	-	−6.07	−3.00	−4.41	0.58
**199**	15.86	25.26	35.87	−139.51	−97.99	−123.03	0.63	0.68	-	−4.98	−3.49	−4.39	0.52
**200**	37.46	31.27	29.79	−139.30	−88.29	−100.49	0.67	0.67	-	−4.97	−3.15	−3.58	0.52
**231**	35.87	24.74	41.80	−145.84	−85.64	−101.52	0.64	0.61	-	−5.60	−3.29	−3.90	0.46
**245**	14.49	24.76	29.38	−137.39	−99.41	−136.65	0.54	0.60	-	−4.43	−3.20	−4.40	0.39
**273**	43.55	27.57	31.78	−143.21	−90.84	−127.36	0.67	0.65	-	−5.30	−3.36	−4.71	0.49
**342**	34.26	27.46	36.14	−141.28	−72.29	−107.30	0.73	0.67	-	−5.65	−2.89	−4.29	0.61
**434**	38.55	29.14	31.53	−138.24	−69.37	−103.72	0.66	0.61	-	−5.31	−2.66	−3.98	0.51
**437**	34.95	26.10	32.63	−143.25	−73.66	−121.15	0.73	0.67	-	−5.73	−2.94	−4.84	0.61
**438**	31.35	25.49	36.05	−143.33	−74.69	−121.91	0.72	0.67	-	−5.73	−2.98	−4.87	0.61
**442**	21.30	29.32	43.77	−147.15	−95.97	−138.47	0.71	0.74	-	−5.88	−3.83	−5.53	0.6
**450**	30.09	25.94	45.40	−123.18	−97.99	−112.14	0.68	0.74	-	−4.92	−3.91	−4.48	0.6
**483**	30.03	22.82	44.95	−112.04	−85.51	−86.760	0.62	0.65	-	−4.48	−3.42	−3.47	0.54
**709**	34.00	26.91	27.69	−143.94	−88.47	− Pr1.28	0.67	0.66	-	−4.96	−3.05	−4.07	0.52
**759**	28.98	31.15	31.87	−119.82	−80.56	−104.55	0.62	0.66	-	−4.60	−3.09	−4.02	0.53
**787**	27.42	24.15	43.73	−120.17	−77.32	−103.20	0.66	0.67	-	−4.62	−2.97	−3.96	0.6
**1015**	16.47	22.09	29.89	−122.68	−93.87	−132.03	0.57	0.64	-	−4.23	−3.23	−4.55	0.48
**1027**	27.07	24.94	40.30	−124.98	−69.30	−80.09	0.63	0.61	-	−4.80	−2.66	−3.08	0.53
**1086**	26.71	22.69	43.52	−170.37	−103.44	−141.82	0.68	0.66	-	−6.08	−3.69	−5.06	0.47
**1184**	24.34	27.89	40.64	−153.42	−97.36	−125.27	0.65	0.67	-	−5.11	−3.24	−4.17	0.49
**1195**	29.74	25.35	43.36	−157.71	−84.13	−124.31	0.75	0.70	-	−6.30	−3.36	−4.97	0.61
**1302**	33.25	22.08	29.37	−143.96	−62.90	−93.11	0.65	0.55	-	−4.79	−2.09	−3.10	0.49
**1350**	33.07	28.87	30.85	−121.54	−87.82	−98.21	0.63	0.67	-	−4.67	−3.37	−3.77	0.53
**1823**	3.18	21.94	31.33	−135.14	−93.79	−110.04	0.56	0.63	-	−4.50	−3.12	−3.66	0.47
**1892**	20.84	17.26	29.68	−132.10	−80.46	−108.72	0.62	0.60	-	−4.89	−2.98	−4.02	0.51
Insecticide	32.12	22.58	37.19	−59.87	−55.07	−163.72	-	-	-	−5.9	−3.6	−3.8	-

**Table 6:** Distribution of diterpenes considered active against *A. gossypii,* with information on the subfamily Nepetoideae (Lamiaceae) listed by tribe, genus, and species (presenting plant data, and occurrence), is available on the SistematX (https://sistematx.ufpb.br) [110].

ID	Popular Name	Subfamily	Tribe	Species	Part of the Plant	Occurrence
**1800**	bistenuifolin I	Nepetoideae	Ocimeae	*Isodon ternifolius*	Aerial parts	China
**1804**	biexcisusin B	Nepetoideae	Ocimeae	*Isodon excisus*	Aerial parts	South Korea
**1809**	bisjaponin A	Nepetoideae	Ocimeae	*Isodon sculponeatus*	Aerial parts	China
				*Isodon japonicus*	Aerial parts	China
**1836**	bistenuifolin L	Nepetoideae	Ocimeae	*Isodon sculponeatus*	Aerial parts	China
**1840**	bistenuifolin E	Nepetoideae	Ocimeae	*Isodon ternifolius*	Aerial parts	China
**1842**	xindongnin N	Nepetoideae	Ocimeae	*Isodon rubescens*	Leaves	China
**1845**	biexcisusin D	Nepetoideae	Ocimeae	*Isodon excisus*	Aerial parts	South Korea
**1854**	staminolactone B	Nepetoideae	Ocimeae	*Orthosiphon stamineus*	Aerial parts	-
**1855**	lushanrubescensin J	Nepetoideae	Ocimeae	*Isodon serra*	-	China
				*Isodon sculponeatus*	Aerial parts	China
				*Isodon rubescens*	Leaves	-
**1910**	siphonol D	Nepetoideae	Ocimeae	*Orthosiphon stamineus*	Aerial parts	Indonesia
**1931**	bistenuifolin K	Nepetoideae	Ocimeae	*Isodon ternifolius*	Aerial parts	China
**1932**	xindongnin M	Nepetoideae	Ocimeae	*Isodon rubescens*	Leaves	China
**1933**	Bistenuifolin M	Nepetoideae	Ocimeae	*Isodon ternifolius*	Aerial parts	China
**1934**	bistenuifolin C	Nepetoideae	Ocimeae	*Isodon ternifolius*	Aerial parts	China
**1936**	inermes B	Nepetoideae	Ocimeae	*Isodon phyllostachys*	Aerial parts	China

**Table 7:** Distribution of diterpenes considered active against *D. melanogaster* with information on the subfamily Nepetoideae (Lamiaceae) listed by tribe, genus, and species, and presenting plant data and occurrence is available on the SistematX (https://sistematx.ufpb.br) [110].

ID	Popular Name	Subfamily	Tribe	Species	Part of the Plant	Occurrence
**21**	isosalvipuberulin	Nepetoideae	Mentheae	*Salvia leucantha*	Aerial parts	Brazil
			Mentheae	*Salvia dugesii*	Leaves and stems	China
			Mentheae	*Salvia tiliifolia*	Aerial parts	Mexico
**44**	salvileucalin A	Nepetoideae	Mentheae	*Salvia leucantha*	Aerial parts	-
**46**	spiroleucantholide	Nepetoideae	Mentheae	*Salvia leucantha*	Aerial parts	Brazil
**47**	dugesin C	Nepetoideae	Mentheae	*Salvia dugesii*	Leaves and stems	China
**51**	dugesin B	Nepetoideae	Mentheae	*Salvia dugesii*	Leaves and stems	China
				*Salvia mexicana*	Aerial parts	Mexico
				*Salvia leucantha*	Aerial parts	China
**67**	salvifaricin	Nepetoideae	Mentheae	*Salvia leucantha*	Aerial parts	Brazil
				*Salvia polystachya*	Aerial parts	Mexico
				*Salvia dugesii*	Leaves and stems	China
**77**	salvifulgenolide	Nepetoideae	Mentheae	*Salvia fulgens*	Aerial parts	Japan
				*Salvia gesneriiflora*	Aerial parts	Mexico
**95**	blepharolide B	Nepetoideae	Mentheae	*Salvia blepharophylla*	Aerial parts	Italy
**111**	salvioccidentalin	Nepetoideae	Mentheae	*Salvia occidentalis*	Aerial parts	Mexico
**131**	salviarin	Nepetoideae	Mentheae	*Salvia splendens*	Aerial parts	China
				*Sa/via divinorum*	Leaves	Mexico
**151**	20-Hydroxydugesin B	Nepetoideae	Mentheae	*Salvia leucantha*	Aerial parts	China
**163**	2-Epi-6,7-dihydrosalviandulin E	Nepetoideae	Mentheae	*Salvia leucantha*	Aerial parts	China
**164**	2-Epi-6,7-dihydrosalviandulin E	Nepetoideae	Mentheae	*Salvia leucantha*	Aerial parts	China
**199**	languiduline	Nepetoideae	Mentheae	*Salvia languidula*	-	Mexico
**200**	salvileucanthsin D	Nepetoideae	Mentheae	*Salvia leucantha*	Aerial parts	China
**231**	dugesin G	Nepetoideae	Mentheae	*Salvia dugesii*	Leaves and stems	China
**245**	6β-butyroxy-3β-hydroxy-6,7-seco-6,20-epoxy-7,1α-olide-entkaur-16-en-15-one	Nepetoideae	Ocimeae	*Isodon japonicus*	Aerial parts	China
**273**	salvileucanthsin A	Nepetoideae	Mentheae	*Salvia leucantha*	Aerial parts	China
**342**	tonalensin	Nepetoideae	Mentheae	*Salvia tonalensis*	Aerial parts	Mexico
**434**	polystachyne E	Nepetoideae	Mentheae	*Salvia polystachya*	Aerial parts	Mexico
**437**	3-*epi*-tilifodiolide	Nepetoideae	Mentheae	*Salvia leucantha*	Aerial parts	China
**438**	3-*epi*-tilifodiolide	Nepetoideae	Mentheae	*Salvia leucantha*	Aerial parts	China
**442**	7,8-Didehydrorhyacophiline	Nepetoideae	Mentheae	*Salvia reflexa*	Leaves	Argentina
**450**	dugesin A	Nepetoideae	Mentheae	*Salvia dugesii*	Leaves and stems	China
**483**	linearolactone	Nepetoideae	Mentheae	*Salvia polystachya*	Aerial parts	Mexico
**709**	salvifolin	Nepetoideae	Mentheae	*Salvia tiliifolia*	Aerial parts	Mexico
**759**	de-*O*-acetylsalvigenolide	Nepetoideae	Mentheae	*Salvia leucantha*	Aerial parts	China
**787**	Salvileucanthsin B	Nepetoideae	Mentheae	*Salvia leucantha*	Aerial parts	China
**1015**	splenolide B	Nepetoideae	Mentheae	*Salvia splendens*	Aerial parts	Italy
**1027**	salviarin	Nepetoideae	Mentheae	*Salvia buchananii*	Aerial parts	Italy
				*Salvia rhyacophila*	Aerial parts	Mexico
				*Salvia reflexa*	Leaves	Argentina
				*Salvia sousae*	Aerial parts	Mexico
**1086**	salvisplendin C	Nepetoideae	Mentheae	*Salvia splendens*	Flowers	Italy
**1184**	salvianduline A	Nepetoideae	Mentheae	*Salvia lavanduloides*	Aerial parts	Mexico
**1195**	salvixalapadiene	Nepetoideae	Mentheae	*Salvia xalapensis*	Aerial parts	Mexico
**1302**	racemosin A	Nepetoideae	Ocimeae	*Isodon henryi*	Leaves	China
**1350**	cardiophyllidin	Nepetoideae	Mentheae	*Salvia cardiophylla*	Aerial parts	-
**1823**	salvilanguiduline A	Nepetoideae	Mentheae	*Salvia languidula*	-	Mexico
**1892**	salvifiline B	Nepetoideae	Mentheae	*Salvia filipes*	Aerial parts	Mexico

**Table 8:** Toxicity evaluation for compounds considered active and multitarget for *A. gossypii*.

ID	Mutagenic	Tumorigenic	Reproductive Effective	Irritant
**1800**	No	No	No	No
**1804**	No	No	No	No
**1809**	No	No	No	High
**1836**	No	No	No	No
**1840**	No	No	No	No
**1842**	No	No	No	No
**1845**	No	No	No	No
**1854**	No	No	No	High
**1855**	No	No	No	High
**1910**	No	No	No	No
**1931**	No	No	No	No
**1932**	No	No	No	No
**1933**	No	No	No	No
**1934**	No	No	No	No
**1936**	High	High	None	High

**Table 9:** Toxicity evaluation of compounds considered active and multitargeting for *D. melanogaster*.

ID	Mutagenic	Tumorigenic	Reproductive Effective	Irritant
**21**	No	No	No	No
**44**	No	No	No	No
**46**	No	No	No	No
**47**	No	No	No	No
**51**	No	No	No	No
**67**	No	No	No	No
**77**	No	No	No	No
**95**	No	No	No	No
**111**	No	No	No	No
**131**	No	No	No	No
**151**	No	No	No	No
**163**	No	No	No	High
**164**	No	No	No	High
**199**	No	No	No	No
**200**	No	No	No	No
**231**	No	No	No	No
**245**	No	No	No	High
**273**	High	High	High	High
**342**	No	No	No	No
**434**	No	No	No	No
**437**	No	No	No	High
**438**	No	No	No	High
**442**	No	No	No	No
**450**	No	No	No	High
**483**	No	No	No	No
**709**	No	No	No	Low
**759**	No	No	No	No
**787**	No	No	No	No
**1015**	No	No	No	No
**1027**	No	No	No	No
**1086**	No	No	No	No
**1184**	No	No	No	No
**1195**	No	No	No	No
**1302**	No	No	No	No
**1350**	No	No	No	No
**1823**	Low	No	No	No
**1892**	Low	No	No	No

**Table 10:** Summaries of the interaction values of the main amino acids for *A. gossipy*.

ID	AChE					
	Tyr135	Tyr133	Trp359	Leu366	Phe409	Val415
**1836**	−14.09	−18.56	−28.50	−5.96	−17.31	−10.38
**1931**	−18.49	−9.49	−43.59	−2.40	−12.61	−2.29
Insecticide	−11.49	−6.28	−24.30	−1.54	−3.34	-
**ID**	**nAChR**					
	**Gln188**	**Ala189**	**Pro190**	**Asp191**	**Ser192**	**Ile195**
**1836**	−11.09	1.09	−29.03	−19.66	−17.34	−12.69
**1931**	−12.00	−6.28	−11.97	−5.38	−17.85	−10.51
Insecticide	−12.41	−5.08	−8.12	−3.13	−8.09	−9.57
**ID**	**Cht**					
	**Trp16**	**Trp87**	**Asn88**	**Arg281**	**Ser282**	**Gln204**
**1836**	−26.47	−32.65	−16.63	−9.30	−7.81	−7.07
**1931**	−30.84	−16.66	−8.34	−6.31	−7.12	-
Insecticide	−35.53	−13.70	−6.83	-	−2.97	-

**Table 11:** Summaries of the interaction values of the main amino acids for *D. melanogaster*.

ID	AChE					
	Tyr71	Gly149	Gly151	Tyr162	Ser238	His480
**1086**	−4.21	−11.39	−6.80	−5.09	−7.60	−11.39
**1195**	−3.23	−4.61	−1.94	-	−3.12	−9.90
Insecticide	-	-	-	-	-	−5.26
**ID**	**nAChR**					
	**Tyr137**	**Trp193**	**Tyr243**	**Lys189**	**Asn138**	**Tyr250**
**1086**	−28.27	−22.45	−26.02	−7.21	−4.75	−4.19
**1195**	−24.17	−15.21	−14.73	−3.68	−12.14	-
Insecticide	−16.26	−16.95	−23.04	-	-	−7.89
**ID**	**Cht**					
	**Trp89**	**Trp349**	**Phe287**	**Trp18**	**Glu131**	**Asp202**
**1086**	−28.39	−23.29	−15.87	−10.29	−9.30	−11.99
**1195**	−20.75	−21.12	−14.26	−4.43	−10.61	−10.42
Insecticide	−25.77	−28.63	−16.71	−11.56	−10.46	−9.41

**Table 12:** Information on the AChE, nAChR, and Cht enzymes for each species selected in the study.

Species	Enzyme	Technique	Insecticide Structure	Insecticide Name	RMSD
*Aphis gossypii*	AChE	Homology	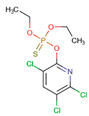	Chlorpyrifos	1.07
*Aphis gossypii*	nAChR	Homology	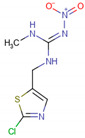	Clothianidin	1.9
*Aphis gossypii*	Cht	Homology	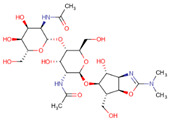	Allosamidin	1.8
*Drosophila melanogaster*	AChE	PDB ID 1DX4	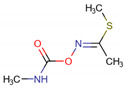	Methomyl	0.32
*Drosophila melanogaster*	nAChR	Homology	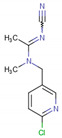	Acetamiprid	1.87
*Drosophila melanogaster*	Cht	Homology	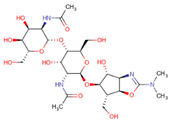	Allosamidin	0.6

**Table 13:** Set of molecules from the ChEMBL databases for each species or genus selected in the study.

Species/Genus	Active Molecules	Inactive Molecules	Total
*Aphis*	91 (≥4.5)	75 (pLC_50_ < 4.5)	166
*Drosophila melanogaster*	105 (≥4.5)	103 (pIC_50_ < 4.5)	208

## Data Availability

The data presented in this study are available on request from the corresponding author.

## Abbreviations

APD	Applicability Domain
FP	False Positives
FN	False Negatives
MCC	Matthews Correlation Coefficient
MVD	Molegro Virtual Docker (MVD) 6.0
PDB	Protein Data Bank
RF	Random Forest
ROC	Receiver Operating Characteristic
RMSD	Mean Square Root Deviation
TN	True Negative
VS	Virtual Screening
VP	True Positive
GHs	Glycosyl hydrolases
TIM	Triosephosphate isomerase
AChE	Acetylcholinesterase
ACh	Acetylcholine
QSAR	Quantitative structure−activity relationship
nAChRs	Nicotinic acetylcholine receptors
mAChRs	Muscarinic acetylcholine receptors
Cht	Chitinase
ADME	Absorption, distribution, metabolism, and excretion
PPV	Positive predicted value
